# Size-Segregated Incense Aerosols Drive ROS–Mitochondrial Dysfunction and Programmed Cell Death Across Human Cell Types

**DOI:** 10.1021/acs.chemrestox.6c00020

**Published:** 2026-02-09

**Authors:** Yi-En Tseng, Ming-Chu Teng, Yu-Siou Huang, Padhmavathi Selvam, Chia-Hsuan Pan, Yuan-Pin Chang, Chia C. Wang, Hsiu-Fang Fan

**Affiliations:** † Institute of Medical Science and Technology, 34874 National Sun Yat-sen University, Kaohsiung 804, Taiwan; ‡ Department of Chemistry, National Sun Yat-sen University, Kaohsiung 804, Taiwan; § Aerosol Science Research Center, National Sun Yat-sen University, Kaohsiung 804, Taiwan

## Abstract

Incense burning is a major indoor source of fine and ultrafine particulate matter (PM), yet the size–chemistry determinants of its cellular toxicity remain underdefined. We characterized aerosols from three commonly used incense types using Aerodynamics Particle Sizer (APS)/Scanning Mobility Particle Sizer (SMPS) for sizing, Micro-Orifice Uniform Deposit Impactor (MOUDI) for size segregation, and water-soluble phase (WP) or organic-phase (OP) extraction to generate incense aerosol extracts (IAEs). Across A549, HEK293T, and SH-SY5Y cells, OP-IAEs from fraction III (0.18–0.10 μm) and IV (<0.10 μm) exhibited the strongest cytotoxicity, oxidative responses, and mitochondrial dysfunction. Type A incense (sandalwood-dominant) IAEs consistently showed the highest potency among the investigated incenses. Mechanistic assays revealed that ultrafine OP-IAEs, elevated intracellular H_2_O_2_, decreased mitochondrial membrane potential (MMP), depleted ATP, and activated apoptosis (caspase-3), pyroptosis (caspase-1), and autophagy-associated pathways. Moreover, ≥80% of all emitted particles were <0.18 μm and were disproportionately enriched in OP constituents across incense types. Collectively, these results identify ultrafine, lipophilic aerosol fractions as key drivers of oxidative–mitochondrial injury and programmed cell death, establishing a size- and phase-resolved framework for assessing incense-related health risks and for guiding exposure mitigation in incense-rich indoor environments.

## Graphical abstract

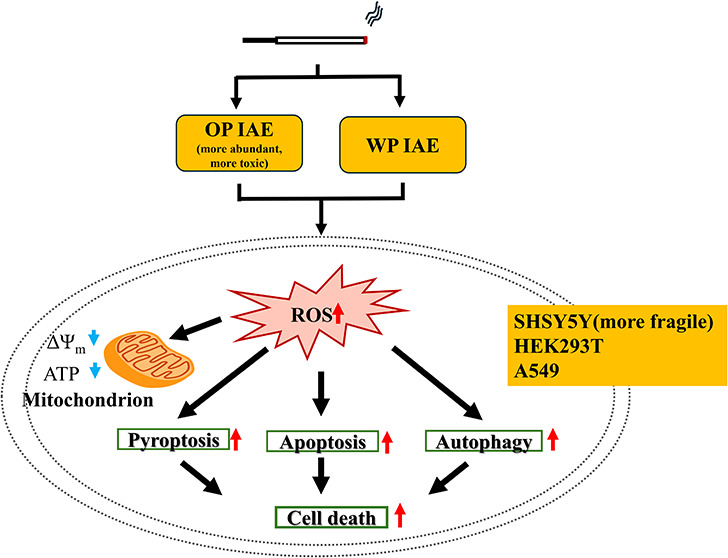

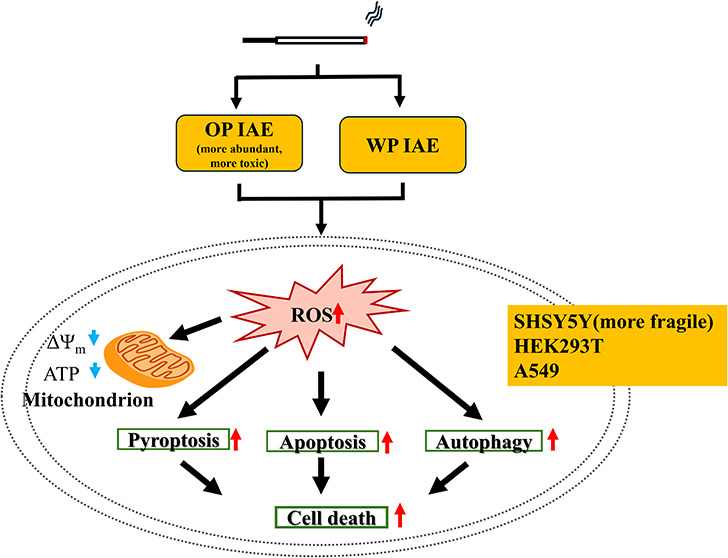

## 1 Introduction

Incense burning is a widespread cultural and domestic practice, particularly in East and Southeast Asia. In addition to its use in religious rituals and ancestor worship, incense is commonly burned indoors for fragrance and deodorizing purposes. Surveys indicate that nearly 80% of Chinese residents burn incense at home daily, and more than 90% have maintained this practice for over two decades, underscoring the magnitude of long-term exposure in the general population.^1^ Incense burning is characterized as a slow, low-temperature, flameless, and incomplete combustion process, with an estimated combustion efficiency of approximately 60–70%.^2,3^ This inefficient combustion generates a complex mixture of gaseous and particulate emissions, commonly referred to as incense smoke. Chemical analyses reveal that incense smoke is a complex mixture containing numerous hazardous substances, including particulate matter (PM), polycyclic aromatic hydrocarbons (PAHs), aldehydes, benzene, toluene, nicotine, volatile organic compounds, and semivolatile organic compounds (SVOCs).^4–6^ Some of these constituents, including auramine O, a common colorant, are classified as potential human carcinogens.^7–10^

Suspended PM is commonly categorized by aerodynamic diameter into three major fractions: ultrafine particles (PM_0_._1_), with diameters ≤0.1 μm; fine particles (PM_2_._5_), ranging from 0.1 to 2.5 μm; and coarse particles (PM_10_), with diameters between 2.5 and 10 μm. Numerous studies have shown that the smaller the particle size, the deeper the penetration into the respiratory system and the greater the associated adverse health effects.^11–14^ Collected PM from incense smoke are dominated by fine and ultrafine particles, with diameters as small as 0.13 μm,^15^ enabling deep penetration into the respiratory tract and potential translocation into systemic circulation.

It has been reported that inhalation of incense smoke induce oxidative stress-mediated respiratory impairment in both animal and in vitro models.^7,16,17^ Notably, emission rates of PM_2_._5_ can exceed 45 mg/g for incense smoke, substantially surpassing the approximately 10 mg/g reported for conventional cigarette smoke.^9,18^ Cardiovascular toxicity has been demonstrated in rodent models is characterized by endothelial dysfunction, cardiac inflammation, oxidative damage, and ultrastructural myocardial alterations, indicating elevated risks for coronary artery disease and stroke.^19,20^ Renal injury, associated with oxidative stress and depletion of antioxidant enzymes, has also been documented in chronically exposed rats.^21^ Furthermore, both cellular and animal models have demonstrated that incense exposure induces oxidative injury and inflammation in brain tissue, disrupts neurodevelopment, and alters gene expression profiles, highlighting its neurotoxic potential.^22,23^ Previous studies have reported that incense smoke induces cytotoxicity and multisystem toxicity through oxidative stress, DNA damage, inflammation, and structural cellular injury.^24,25^ Incense smoke exposure increases intracellular reactive oxygen species (ROS), reduce antioxidant enzyme activity, and promotes apoptosis, all of which are mechanisms implicated in tumorigenesis and chronic diseases.^17,24^

Despite increasing evidence of incense smoke–induced toxicity, critical knowledge gaps remain in understanding its mechanistic pathways and enabling systematic comparisons across studies. Previous investigations have typically focused on a single organ system—most commonly the respiratory system—or examined only a limited number of incense products. In addition, substantial methodological heterogeneity exists, including differences in whether the gaseous phase alone or whole smoke (gaseous components plus suspended PM) is collected, as well as variability in extraction media (e.g., phosphate-buffered saline, cell culture medium, or organic solvents). These inconsistencies have hindered clear identification of the size- and chemistry-resolved determinants of toxicity and have limited cross-study comparability. To overcome these limitations, the present study introduces a standardized and integrative experimental framework that specifically targets suspended incense-derived PM (hereafter referred to as “aerosols”). Building upon established methodologies,^26,27^ we precisely control incense aerosol generation, perform aerodynamic size-segregated particle collection, and apply parallel aqueous and organic extraction strategies to isolate water-soluble and organic-soluble toxic constituents. Furthermore, by employing three human-derived cell lines representing distinct target organs—Human nonsmall cell lung carcinoma (A549), human embryonic kidney (HEK293T), and human neuroblastoma (SH-SY5Y)—we directly assess cell type–specific vulnerabilities to incense aerosol extracts (IAE). In parallel, aerosols derived from three commonly used incense types—sandalwood-dominant (Type A), agarwood-dominant (Type B), and binchotan charcoal–based (Type C)—are systematically compared, enabling evaluation of compositional variability and its impact on cellular outcomes. Collectively, this multidimensional, size-, chemistry-, cell type–, and product-resolved framework distinguishes our study from prior work and establishes a unified platform for systematic comparison of incense aerosol toxicity across exposure scenarios.

Using this standardized and integrative platform, we demonstrate that incense aerosols with diameters smaller than 0.18 μm exhibit the highest biological potency, likely attributable to their enrichment of surface-bound toxicants and enhanced cellular internalization. In addition, organic-phase (OP) extracts—enriched in lipophilic PAHs and SVOCs—elicit more pronounced oxidative stress and mitochondrial dysfunction than their water-soluble counterparts. These upstream stress responses subsequently converge on multiple programmed cell death pathways, including apoptosis, pyroptosis, and autophagy, with SH-SY5Y neuronal cells displaying markedly greater vulnerability than A549 lung epithelial and HEK293T renal cells. Importantly, beyond these specific findings, the platform established in this study enables systematic, size- and chemistry-resolved comparison of the cytotoxicity of complex aerosol constituents across different biological targets and product types. This versatile framework provides a robust basis for mechanistic toxicology studies and offers actionable insights for indoor air quality assessment and exposure mitigation strategies.

## 2 Materials and Methods

### 2.1 Aerodynamics Particle Sizer (APS) and Scanning Mobility Particle Sizer (SMPS) Measurement of Incense Aerosol

The size distribution and number concentration of incense combustion aerosols were characterized using an APS (TSI Inc.) and a SMPS (model 3936, TSI Inc.) which together cover particle sizes from the nanometer to micrometer range. Incense sticks from three types incenses. Type A (sandalwood-dominant), Type B (agarwood-dominant), and Type C (binchotan charcoal-based) details listed in Table 1 were cut to 1 cm combustible length, ignited, and placed in a custom-built combustion chamber. After a 30 s stabilization period, aerosols were sampled for 60 s. Each measurement was repeated three independent times per incense type, with the chamber purged for 5 min between runs to avoid cross-contamination.

**Table 1:**
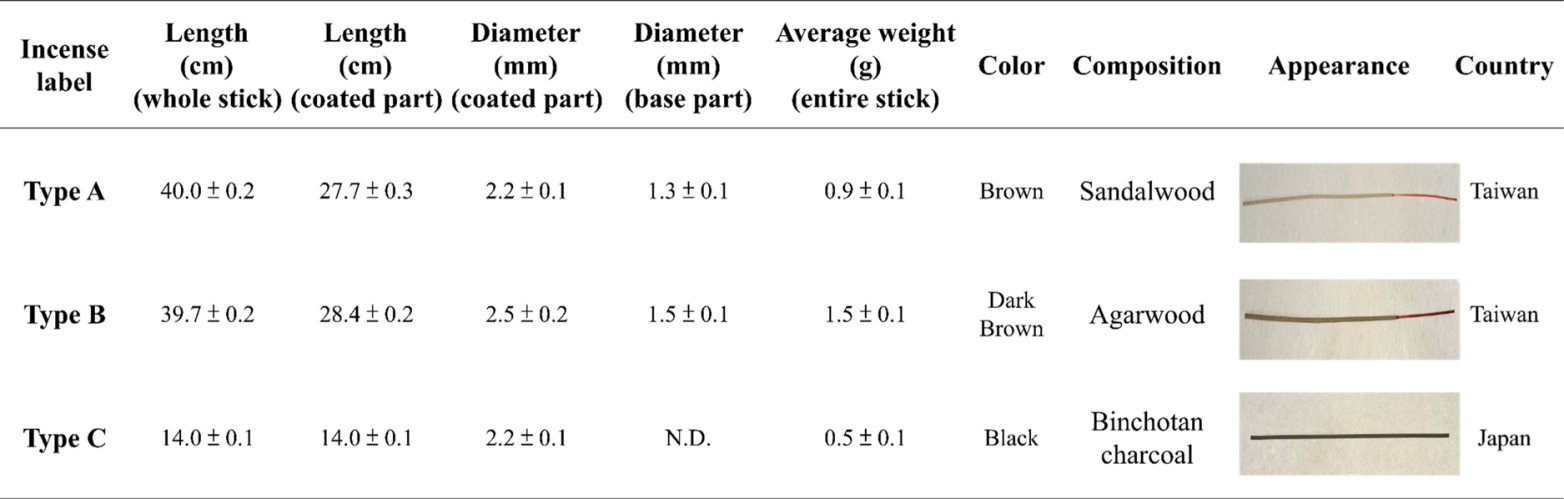
Physical Characteristics of Investigated Incense Sticks (*n* = 3 for Each Type of Incense)

APS measurements were conducted at a flow rate of 5 L/min for 60 s to assess particles in the 0.5–20 μm range [Figure 1A­(i)]. SMPS measurements were performed at a flow rate of 0.3 L min^–1^ to resolve submicron and ultrafine particles [Figure 1A­(ii)]. To prevent signal saturation during SMPS analysis, aerosol concentration was reduced using a HEPA filtration bypass while maintaining constant flow. Combined APS–SMPS data provided a comprehensive profile of aerosol number concentration and modal size distributions.

**Figure 1:**
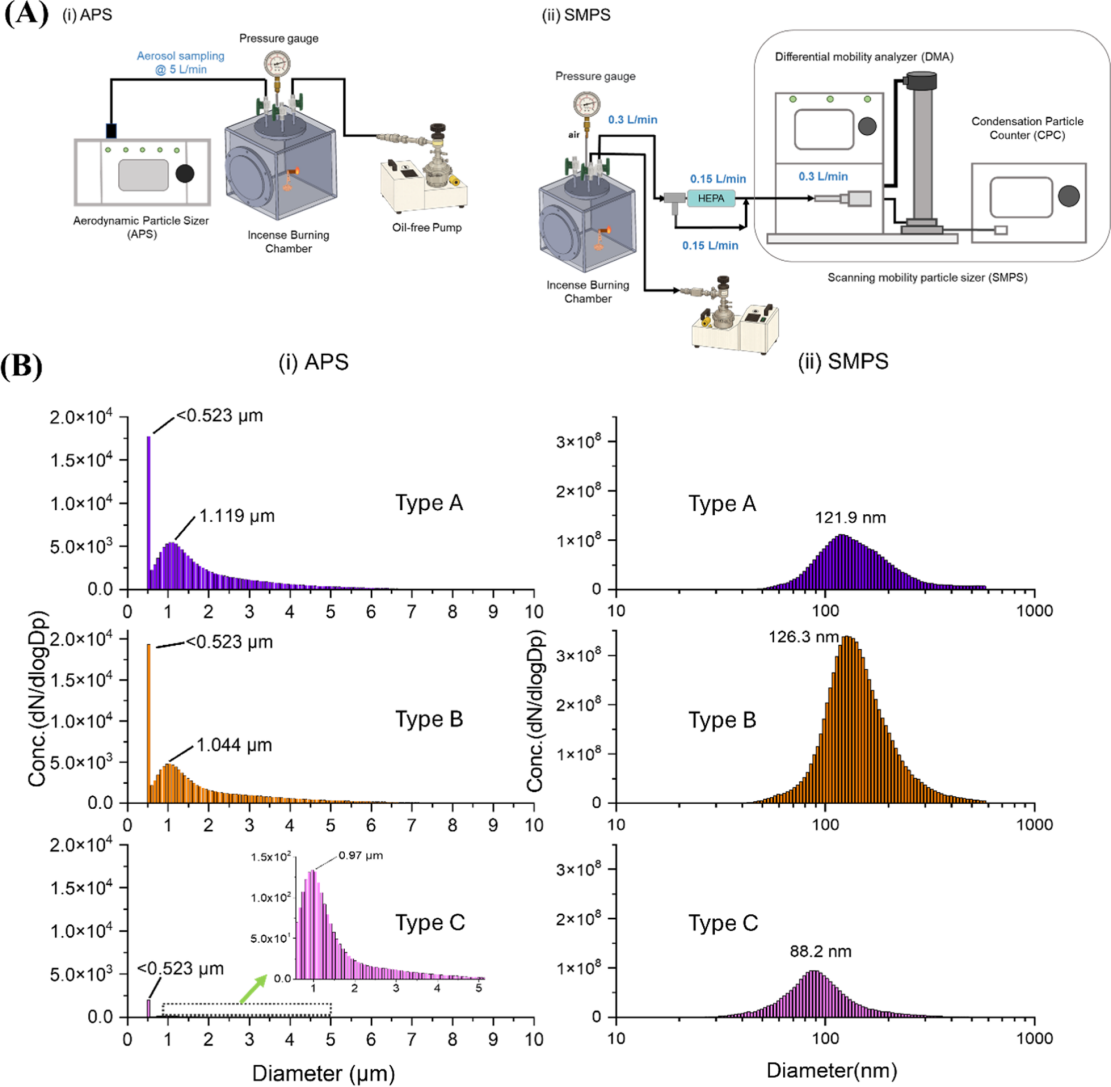
(**A**) Schematic diagram of the measurement procedures using (i) an APS and (ii) a SMPS to characterize the size distribution of collected incense aerosols. (**B**) Representative results of (i) APS and (ii) SMPS measurements for Type A, Type B, and Type C incense aerosols.

### 2.2 Incense Aerosol Collection and Preparation of Incense Aerosol Extract

Incense aerosols were collected using a Micro-Orifice Uniform Deposit Impactor (MOUDI-II) Impactor (model 120 MOUDI II Impactor, USA), operated at a flow of 30 m^3^ min^–1^ (GAST,1023–101Q-SG608X). Aerosols were deposited onto PTFE filters (Finetech, M-PTFE047N010O) across 11 aerodynamic size stages (18–10 μm to <0.056 μm) which are Stage 1:18–10 μm, stage 2:10–5.6 μm, stage 3:5.6–3.2 μm, stage 4:3.2–1.8 μm, stage 5:1.8–1 μm, stage 6:1–0.56 μm, stage 7:0.56–0.32 μm, stage 8:0.32–0.18 μm, stage 9:0.18–0.10 μm, stage 10:0.10–0.056 μm, stage 11: <0.056 μm.

For following analyses, samples were pooled into four size fractions: Fraction I (18–0.56 μm), Fraction II (0.56–0.18 μm), Fraction III (0.18–0.10 μm), and Fraction IV (<0.10 μm). Each pooled fraction was weighed to determine mass distribution. IAE were prepared following our previously reported protocols^26,27^ using ultrapure water to obtain water-soluble phase (WP) IAE and dimethyl sulfoxide (DMSO) to obtain OP IAE. Extracts were filtered, aliquoted, and stored at −80 °C (Figure 2).

**Figure 2:**
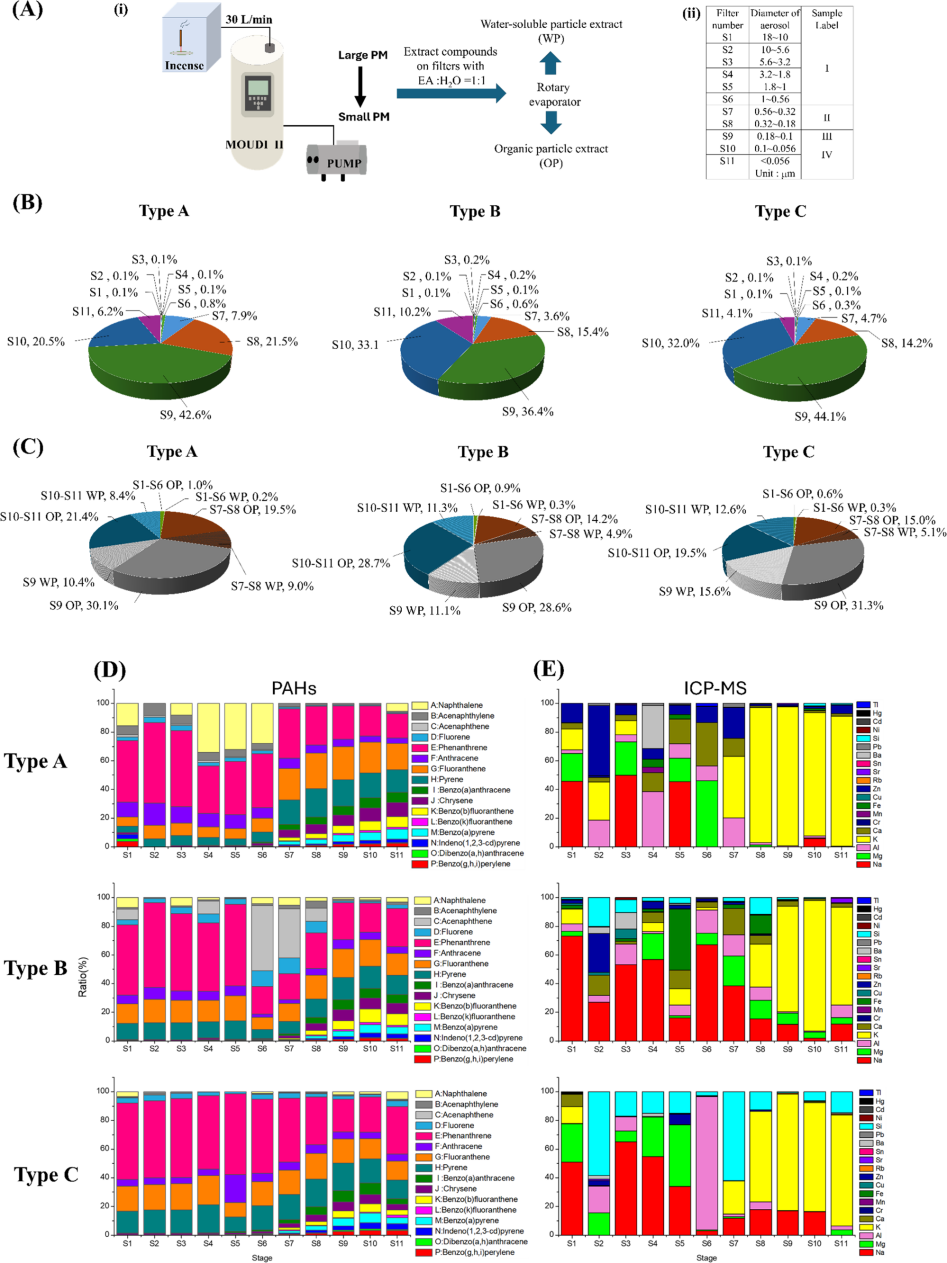
(A) Workflow for incense aerosol collection and extraction, including the sample classification table. (B) Mass distribution of incense aerosol across particle size fractions. (C) Mass distribution of IAEs across particle size fractions. (D) PAHs profiles in OP IAE by GC–MS/MS. (E) Elemental analysis of WP IAEs by ICP–MS.

### 2.3 Chemical Characterization of Size-Segregated Incense Aerosols

OP IAE were analyzed for the U.S. EPA specified 16 environmentally relevant priority PAHs using gas chromatography–tandem mass spectrometry (GC–MS/MS; Agilent 7890A/7000D) operated in multiple-reaction monitoring (MRM) mode with a 30 m Zebron GC column ZB-PAH-EU column (Figure 2D). WP IAE were analyzed for metal ions using inductively coupled plasma mass spectrometry (ICP–MS) (Figure 2E). Chemical characteristics analyses were performed in a size-segregated manner to link aerosol composition with biological outcomes.

### 2.4 Cell Culture and Treatment

HEK293T cells, A549 cells, and SH-SY5Y cell lines were cultured in high-glucose Dulbecco’s modified Eagle’s medium (DMEM, Gibco, 11,965–084) supplemented with 10% (v/v) fetal bovine serum (FBS, Corning, 35–010-CV) and 1% penicillin/streptomycin (Gibco, 15,140–122). Cells were maintained in 10 cm culture dishes (α-plus, 16,203–1SS) at 37 °C in a saturated humidity atmosphere containing 95% air and 5% CO_2_.

### 2.5 Cell Viability Assay

HEK293T cells, A549 cells, and SH-SY5Y cells were seeded at a density of 3.5 × 10^4^ cells/well in 96-well plates (α-plus, 116,196–1SS) and treated with various concentrations of WP IAE and OP IAE obtained from three different burning incenses for 24 h. Cell viability was assessed using the MTT (3-(4,5-dimethylthiazole-2-yl)-2,5-diphenyltetrazolium bromide) assay (Alfa Aesar, L11939). After 24 h of IAE treatment, the medium was removed, and cells were washed with phosphate buffers (137 mM NaCl, 2.7 mM KCl, 10 mM Na_2_HPO_4_, 1.8 mM KH_2_PO_4_). Fresh DMEM containing MTT (0.5 mg/mL) was added, and cells were incubated for 4 h at 37 °C in a humidified atmosphere of 95% air and 5% CO_2_. After that, the solution was removed, and cells were washed with phosphate buffer (137 mM NaCl, 2.7 mM KCl, 10 mM Na_2_HPO_4_, 1.8 mM KH_2_PO_4_). The formazan formed was solubilized in 300 μL of DMSO (Cyrusbioscience, 101–67–68–5). Absorbance was measured at 570 nm using a microplate reader (SpectraMax i3, Molecular Devices). Each assay condition was assessed in at least three independent experiments. Statistical significance between groups was determined using paired *t*-tests.

### 2.6 Measurement of Intracellular ROS

HEK293T cells, A549 cells, and SH-SY5Y cells were seeded at a density of 3.5 × 10^4^ cells/well in 96-well plates (α-plus, 116,196–3SB). For measurement of intracellular hydrogen peroxide (H_2_O_2_), OxiVision Green hydrogen peroxide sensor (AAT Bioquest, 11,503) was preincubated with cells for 1 h before IAE obtained from three different burning incense treatments for an additional 80 min at 37 °C. Treatment concentrations were normalized based on cell sensitivity determined in prior cytotoxicity assays: SH-SY5Y cells were treated with 200 mg/mL OP IAE (due to lower IC_50_ values), while HEK293T and A549 cells were treated with 250 mg/mL OP IAE. Fluorescence intensity inside the cells was quantified using a microplate reader (Molecular Devices, SpectraMax iD3) with an excitation wavelength of 490 nm and an emission wavelength of 525 nm. Each assay condition was assessed in at least three independent experiments. Statistical significance between groups was determined using paired *t*-tests.

### 2.7 Assessment of Mitochondrial Membrane Potential (MMP) Assay

HEK293T cells, A549 cells, and SH-SY5Y cells were plated at a density of 3.5 × 10^4^ cells/well in 96-well plates (α-plus, 116,196–3SB). For measurement of MMP, cells were preincubated with 100 μL of JC-1 (Abcam, ab113850) for 10 min. Then cells were washed with 1X dilution buffer twice before treatment with IAE obtained from three different burning incenses (SH-SY5Y: 200 μg/mL OP IAE; HEK293T and A549:250 μg/mL for OP IAE) for an additional 150 min at 37 °C. OP IAEs were selected for mechanistic assays due to their significantly higher cytotoxicity and oxidative activity. Fluorescence was then measured using a microplate reader with dual excitation/emission settings for monomeric (475 nm/550 nm) and oligomeric (535 nm/590 nm) JC-1 forms. Each assay condition was assessed in at least three independent experiments. Statistical significance between groups was determined using paired *t*-tests.

### 2.8 ATP Quantification Assay

HEK293T cells, A549 cells, and SH-SY5Y cells were plated at a density of 3.5 × 10^4^ cells/well in 96-well plates (α-plus, 116,196–3SB). For measurement of ATP level, cells were treated with IAE obtained from three different burning incenses (SH-SY5Y: 200 μg/mL OP IAE; HEK293T and A549:250 μg/mL for OP IAE) for 150 min at 37 °C. Then 50 μL of detergent (supplied by the vendor) was added and shaken for 5 min. Later, 50 μL of substrate solution (provided by the vendor) was added and shaken for 5 min, followed by an additional 10 min of waiting in the dark. Luminescence intensity was measured with the microplate reader. Each assay condition was assessed in at least three independent experiments. Statistical significance between groups was determined using paired *t*-tests.

### 2.9 Capspase-1/-3 Activity Detection

Cells (1.0 × 10^6^) were treated with IAE obtained from three different types of incenses (at the indicated concentrations) for 24 h at 37 °C. After treatment with IAE obtained from three different types of incenses (at the indicated concentrations), cells were lysed using 50 μL of lysis buffer provided by the manufacturer. Protein concentrations were determined using the Bradford method (Scientific Biotech Corp, BR01–500). The activities of caspase-1 and caspase-3 were detected using caspase activity assay kits, ab273268 (Abcam) and K106–25 (BioVision), respectively. Samples containing 150 μg of total protein were incubated with 50 μL of 2× reaction buffer (supplied by the manufacturer) and 5 μL of 4 mM YVAD-pNA/DEVD-pNA substrate (200 μM final concentration) for 2 h at 37 °C. Absorbance at 400 nm was recorded using a microplate reader (Molecular Devices, SpectraMax iD3). Percentage changes in caspase-1/-3 activity were calculated based on OD_400_ values. Each assay condition was assessed in at least three independent experiments. Statistical significance between groups was determined using paired *t*-tests.

### 2.10 Autophagy Activity Detection Assay

HEK293T cells, A549 cells, and SH-SY5Y cells were plated at a density of 3.5 × 10^4^ cells/well in 96-well plates (α-plus, 116,196–3SB). Autophagy activity was assessed using a fluorescence autophagy assay kit (Abcam, ab139484). Cells were treated with IAE obtained from three different types of incenses (at the indicated concentrations) for 4 h at 37 °C. Post-treatment, cells were incubated with the detection reagent (supplied by the vendor) for 30 min, followed by replacement with 100 μL of 1× assay buffer. Fluorescence intensity was measured using a microplate reader (Molecular Devices, SpectraMax iD3) with excitation at 480 nm and emission at 530 nm. Each assay condition was assessed in at least three independent experiments. Statistical significance between groups was determined using paired *t*-tests.

### 2.11 Statistical Analysis

Data are presented as mean ± standard deviation (SD) from at least three independent experiments. All statistical analyses were performed using OriginPro 2023b (OriginLab Corporation, Northampton, MA, USA). To assess the main effects and interactions between multiple variables (e.g., cell type, incense type, and aerosol fraction), data were analyzed using Two-way or Three-way Analysis of Variance (ANOVA) as appropriate. Following ANOVA, significant differences were determined using Tukey’s post hoc test for multiple comparisons. For simple comparisons between two specific groups, paired *t* tests were used. Differences were considered statistically significant at *p* < 0.05.

## 3 Results

### 3.1 Analysis of Physical Characteristics of Incense Aerosols

The particle number concentration and size distribution of aerosols generated from three types of incense were measured using APS and SMPS (Figure 1). APS measurements showed that aerosols from all incense types were predominantly distributed in the submicron size range (<0.5 μm), with minimal contribution from coarse particles. Type B incense exhibited an additional accumulation mode near ∼1.17 μm, whereas Type A and Type C incenses showed unimodal distributions dominated by submicron particles. SMPS analysis further resolved the ultrafine fraction and revealed distinct modal diameters among incense types. Type A incense generated a unimodal distribution centered around at approximately 122 nm. Type B incense generated similarly high particle numbers but with a broader distribution peaking near ∼127 nm. In contrast, Type C incense emitted relatively fewer particles, with a modal diameter centered at ∼88 nm Integration of the number–size distributions indicated that total particle number emissions were highest for Type B incense, followed by Type A incense, and lowest for Type C incense. Collectively, these measurements demonstrate that incense combustion produces abundant ultrafine aerosols with incense-specific size profiles.

### 3.2 Mass Distribution of Size-Segregated Incense Aerosol

Incense aerosols were collected using a MOUDI-II impactor and grouped into four aerodynamic size fractions (Figure 2A). Gravimetric analysis showed that the majority of aerosol mass was associated with submicron and ultrafine fractions (Figure 2B). For all incense types, particles smaller than 0.18 μm (Fractions III and IV) accounted for more than 80% of the total collected mass. Type A incense showed the highest mass contribution in Fraction III (0.18–0.10 μm; 42.6%), followed by Fraction II (0.56–0.18 μm; 29.4%) and Fraction IV (<0.10 μm; 26.7%), with minimal mass in Fraction I (>0.56 μm; 1.3%). Type B incense exhibited a broader mass distribution, with Fraction IV contributing the largest proportion (43.3%), followed by Fraction III (36.4%) and Fraction II (19.0%). Type C incense displayed a similar pattern, with dominant contributions from Fraction III (44.1%) and Fraction IV (36.1%). These results confirm that incense aerosol predominantly generates ultrafine and nanoparticle aerosols.

### 3.3 Chemical Characteristics of Incense Aerosols

WP IAE and OP IAE were prepared from each size fraction (Figure 2C). Recovery yields approached 100%, indicating that extract mass accurately reflected collected aerosol mass (Table 2). Across all incense types, OP IAE was enriched in Fractions III and IV, whereas WP IAE contributed comparatively less mass in these fractions. Chemical analysis of OP IAE showed the presence of multiple PAHs across all size fractions (Figure 2D). Phenanthrene, fluoranthene, and pyrene were consistently among the most abundant PAHs detected. Analysis of WP IAE revealed the presence of several metal ions, with potassium being the dominant species across all incense types and size fractions (Figure 2E). Higher molecular weight and complicated PAHs appear more abundant in fraction with smaller particle diameter across three investigated incenses. These chemical profiles demonstrate size- and chemistry-dependent differences in aerosol composition.

**Table 2:** Collection and Extraction Yield of Incense Aerosols

Incense label	Fraction	Diameter of aerosol (μm)	Recovery rate (%)	Residue rate (%)
**Type A**	I	>0.56	99.93	0.07
	II	0.56–0.18	100.24	–0.24
	III	0.18–0.10	100.13	–0.13
	IV	<0.10	100.10	–0.10
**Type B**	I	>0.56	91.43	8.57
	II	0.56–0.18	90.68	9.32
	III	0.18–0.10	96.38	3.62
	IV	<0.10	93.16	6.84
**Type C**	I	>0.56	106.36	–6.36
	II	0.56–0.18	96.92	3.08
	III	0.18–0.10	96.99	3.01
	IV	<0.10	94.75	5.25

### 3.4 Cytotoxicity Effects of Incense Aerosol Extracts

The cytotoxic effects of WP IAE and OP IAE were evaluated in SH-SY5Y, HEK293T, and A549 cells using the MTT assay (Figure 3; Table 3). Across all incense types and cell lines, OP IAE exhibited substantially lower IC_50_ values than WP IAE, indicating greater cytotoxic potency. A clear size-dependent trend was observed. For all incense types, cytotoxicity increased with decreasing particle size, with Fractions III and IV consistently producing the lowest IC_50_ values.

**Figure 3:**
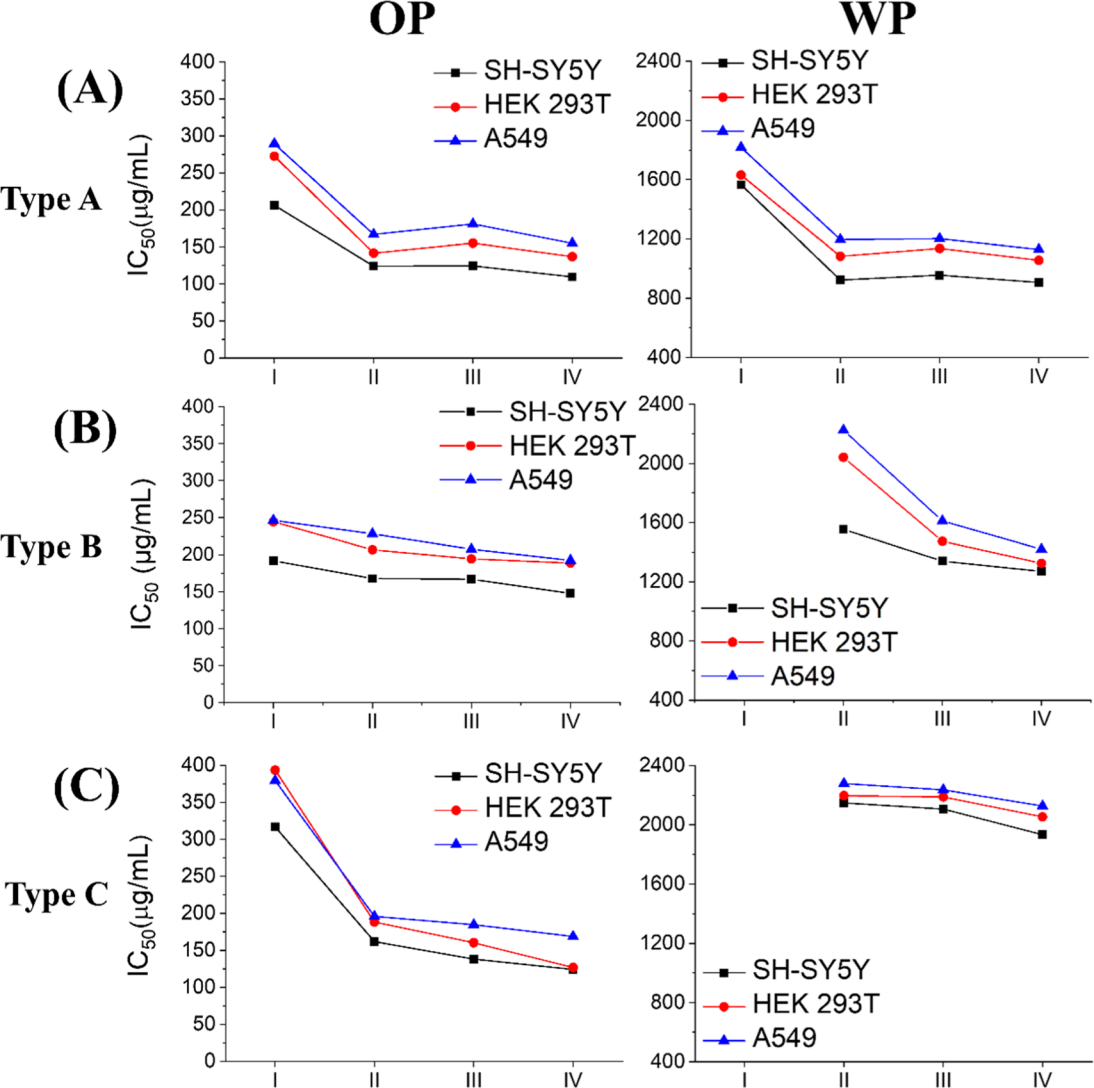
IC_50_ values of IAE-induced cytotoxicity on three human cell lines—SH-SY5Y, HEK293T, and A549—based on three independent experiments. Results are shown for the OP and WP IAE of (A) Type A incense, (B) Type B incense, and (C) Type C incense. I–IV denote aerosol particle size fractions obtained by MOUDI, as listed in Figure 2A.

**Table 3:** IC_50_ Values of Size-Fractionated Incense Aerosols in Three Different Cell Lines

		IC_50_ (μg/mL)					
		SH-SY5Y		HEK293T		A549	
		OP	WP	OP	WP	OP	WP
**I (>0.56 μm)**	Type A	206.4	1565.5	272.7	1631.2	289.6	1817.9
	Type B	215.6	N.D.	244.5	N.D.	246.5	N.D.
	Type C	316.8	N.D.	393.6	N.D.	379.4	N.D.
**II (0.56–0.18 μm)**	Type A	124.0	921.6	141.6	1082.5	167.0	1195.0
	Type B	167.7	1554.0	206.7	2041.8	228.2	2224.0
	Type C	161.7	2148.5	188.2	2197.1	195.8	2278.9
**III (0.18–0.1 μm)**	Type A	124.4	954.7	155.1	1135.6	181.0	1201.7
	Type B	166.9	1339.7	194.3	1473.9	207.6	1611.4
	Type C	138.0	2106.7	160.2	2188.8	184.6	2236.2
**IV (<0.1 μm)**	Type A	109.5	905.5	137.0	1055.7	154.9	1128.3
	Type B	147.6	1269.3	188.7	1324.2	192.3	1419.6
	Type C	124.0	1933.2	126.7	2054.0	168.6	2127.7

For Type A incense, OP IAE showed the greatest cytotoxicity overall. In SH-SY5Y cells, IC_50_ values decreased from 206.4 μg/mL (Fraction I) to 109.5 μg/mL (Fraction IV). Similar reductions were observed in HEK293T (272.74 to 137.0 μg/mL) and A549 (289.6 to 154.9 μg/mL). WP IAE were far less potent, with IC_50_ values ranging from 1565.3–905.5 μg/mL in SH-SY5Y, 1631.2–1055.7 μg/mL in HEK293T, and 1817.9–1128.3 μg/mL in A549. These results establish Type A incense OP IAE, particularly from ultrafine and nanoparticle fractions, as the most cytotoxic among the incense types.

For Type B incense, OP IAE displayed intermediate potency. In SH-SY5Y, IC_50_ values declined from 215.6 μg/mL (Fraction I) to 147.6 μg/mL (Fraction IV). Corresponding decreases were observed in HEK293T (244.5 to 188.7 μg/mL) and A549 (246.5 to 192.3 μg/mL). Due to the lower extract mass obtained for Fraction I WP-IAE, IC_50_ values could not be determined for this fraction. In Fractions II–IV, WP IAE yielded much higher IC_50_ values, ranging from 1269.3 μg/mL in SH-SY5Y, 1324.2 μg/mL in HEK293T, and 1419.6 μg/mL in A549.

For Type C incense, OP IAE was the least cytotoxic. In SH-SY5Y, IC_50_ values decreased from 316.8 μg/mL (Fraction I) to 124.0 μg/mL (Fraction IV). Similar trends were observed in HEK293T (393.6 to 126.7 μg/mL) and A549 (379.4 to 168.6 μg/mL). WP IAE again displayed minimal toxicity. Because of the limited extract mass for Fraction I WP-IAE, IC_50_ values could not be determined. In Fractions II–IV, IC_50_ values remained at very high concentrations—2148.5–1933.2 μg/mL in SH-SY5Y, 2197.1–2054.0 μg/mL in HEK293T, and 2278.9–2127.7 μg/mL in A549.

Among the three types of incense, Type A incense OP IAE displayed the strongest cytotoxicity, followed by Type C and Type B incenses. In contrast, for WP IAE, Type A incense remained the most cytotoxic, followed by Type B, while Type C was the least toxic. SH-SY5Y cells exhibited the lowest IC_50_ values across most conditions, whereas HEK293T and A549 cells showed comparatively higher tolerance. WP IAE generally produced minimal cytotoxicity, with IC_50_ values exceeding 1 mg mL^–1^ in most cases.

### 3.5 Induction of Intracellular ROS

Because OP IAE was approximately 3-fold more abundant than WP IAE (Figure 2C), OP IAE–induced ROS generation was assessed to represent the oxidative potency of incense aerosols. Intracellular H_2_O_2_ levels were quantified following exposure to size-resolved IAE (Figure 4). Three-way ANOVA confirmed significant main effects for cell type, incense type, and IAE fraction (*p* < 0.0001), with significant two-way interactions (*p* < 0.01) but no significant three-way interaction. Post hoc analysis identified Incense Type A as the most potent inducer of ROS. Among the tested models, SH-SY5Y cells exhibited the highest sensitivity, yielding greater ROS responses than A549 or HEK293T cells despite lower exposure concentrations. Oxidative stress induction was strongly size-dependent; ROS levels increased as particle size decreased, with the ultrafine Fraction IV eliciting the highest response across all groups. This toxicity hierarchy (Fraction IV≅III > II > I) and incense ranking (Type A > C > B) consistently aligned with the IC_50_ trends observed in cell viability assays (Figure 3).

**Figure 4:**
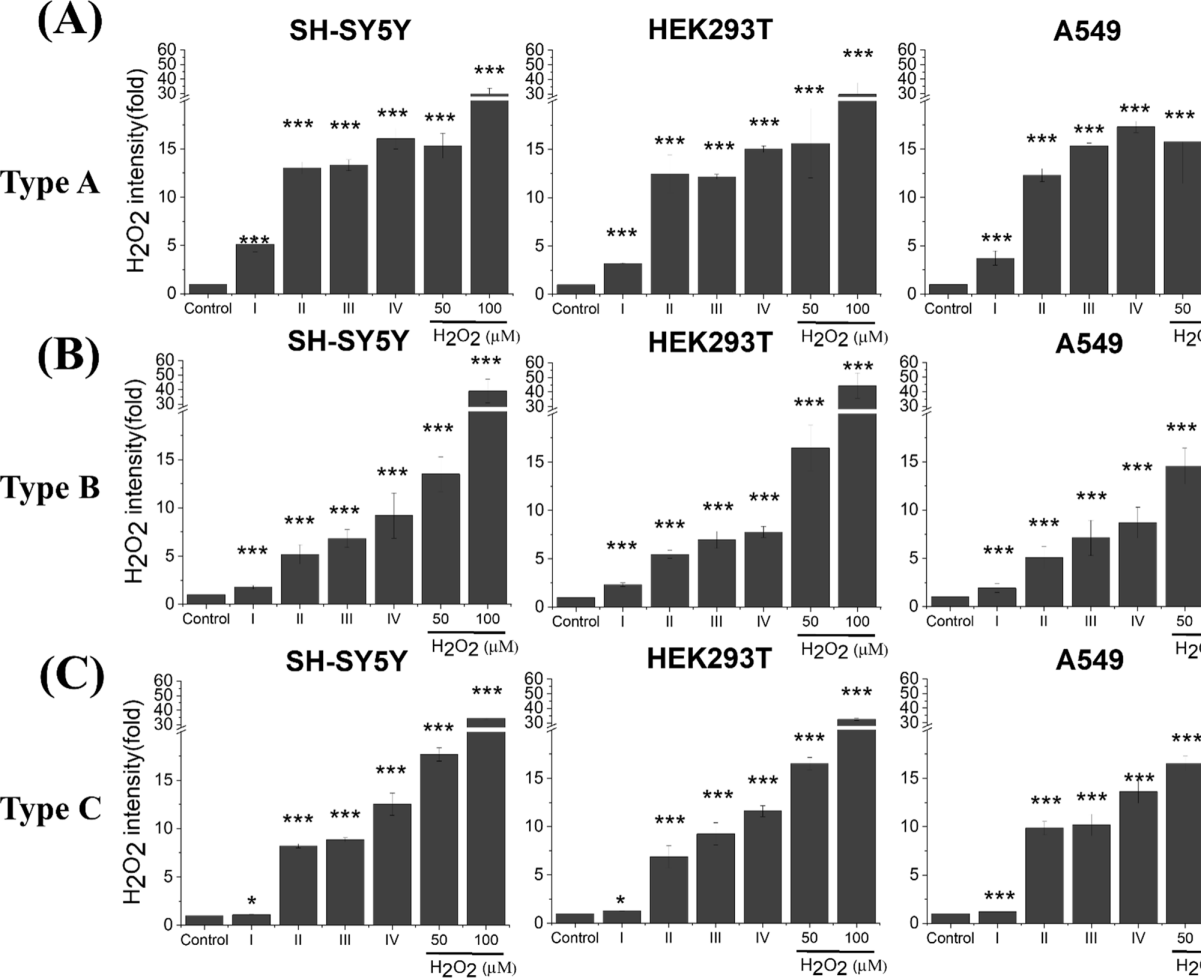
Intracellular hydrogen peroxide (H_2_O_2_) generation in living SH-SY5Y, HEK293T, and A549 cells following treatment with IAEs from­(A) Type A incense, (B) Type B incense, and (C) Type C incense. I–IV indicate aerosol particle size fractions (see Figure 2A). Data represents mean ± SD from three independent experiments. Statistical significance: *p* < 0.05 (*), *p* < 0.01 (**), *p* < 0.005 (***).

### 3.6 Effects of Incense Aerosol Extracts on Mitochondrial Integrity via Membrane Potential Loss and ATP Depletion

Following the observation of robust ROS induction, we investigated whether IAE-mediated oxidative stress translated into mitochondrial dysfunction and bioenergetic impairment. Due to their higher abundance and oxidative potency, OP-IAEs were selected for these analyses. MMP ΔΨm and intracellular ATP levels were assessed via JC-1 staining and bioluminescence assays, respectively (Figure 5). Three-way ANOVA revealed significant main effects for cell type, incense type, and IAE fraction on both ΔΨm and ATP generation (*p* < 0.05), with significant two- and three-way interactions observed across all factors. Post hoc analysis showed that Incense Type A induced the most severe mitochondrial depolarization and ATP depletion. This dysfunction was strongly size-dependent: Fraction IV elicited the most pronounced reduction in ΔΨm and ATP levels, whereas Fraction I caused minimal impairment.

**Figure 5:**
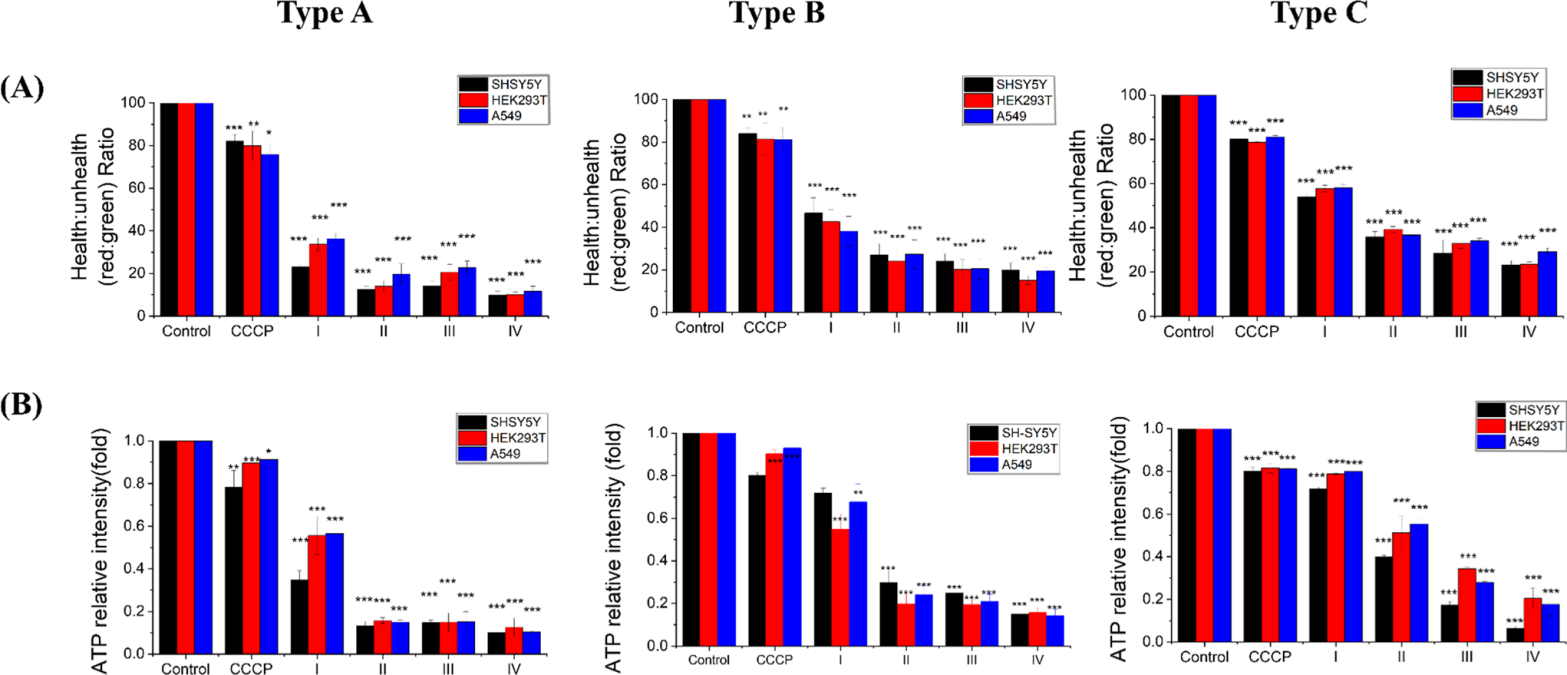
Effects of IAEs from Type A incense, Type B incense, and Type C incense on (A) MMP polarization and (B) ATP production in SH-SY5Y (black), HEK293T (red), and A549 (blue) cells. I–IV indicate aerosol particle size fractions (see Figure 2A). Data represents mean ± SD from three independent experiments. Statistical significance: *p* < 0.05 (*), *p* < 0.01 (**), *p* < 0.005 (***).

Consistent with the ROS data, SH-SY5Y cells demonstrated the greatest mitochondrial vulnerability, particularly when exposed to Type A IAE in the smaller particle fractions. The observed collapse of ΔΨm (Figure 5A) closely mirrored the significant reduction in cellular ATP (Figure 5B), suggesting that fine-fraction incense aerosols—specifically from Type A—induce severe energetic dysfunction by disrupting mitochondrial integrity.

### 3.7 Activation of Programmed Cell Death

Given the robust cytotoxicity, ROS generation, and mitochondrial dysfunction observed across IAE Fractions II–IV (Figures 2–5), Fraction III OP IAE—the most abundant fraction—was selected to assess the activation of programmed cell death (PCD) pathways. As shown in Figures 6, 7 and 8, Fraction III OP IAE from all three incense types significantly activated multiple PCD pathways in SH-SY5Y, HEK293T, and A549 cells.

**Figure 6:**
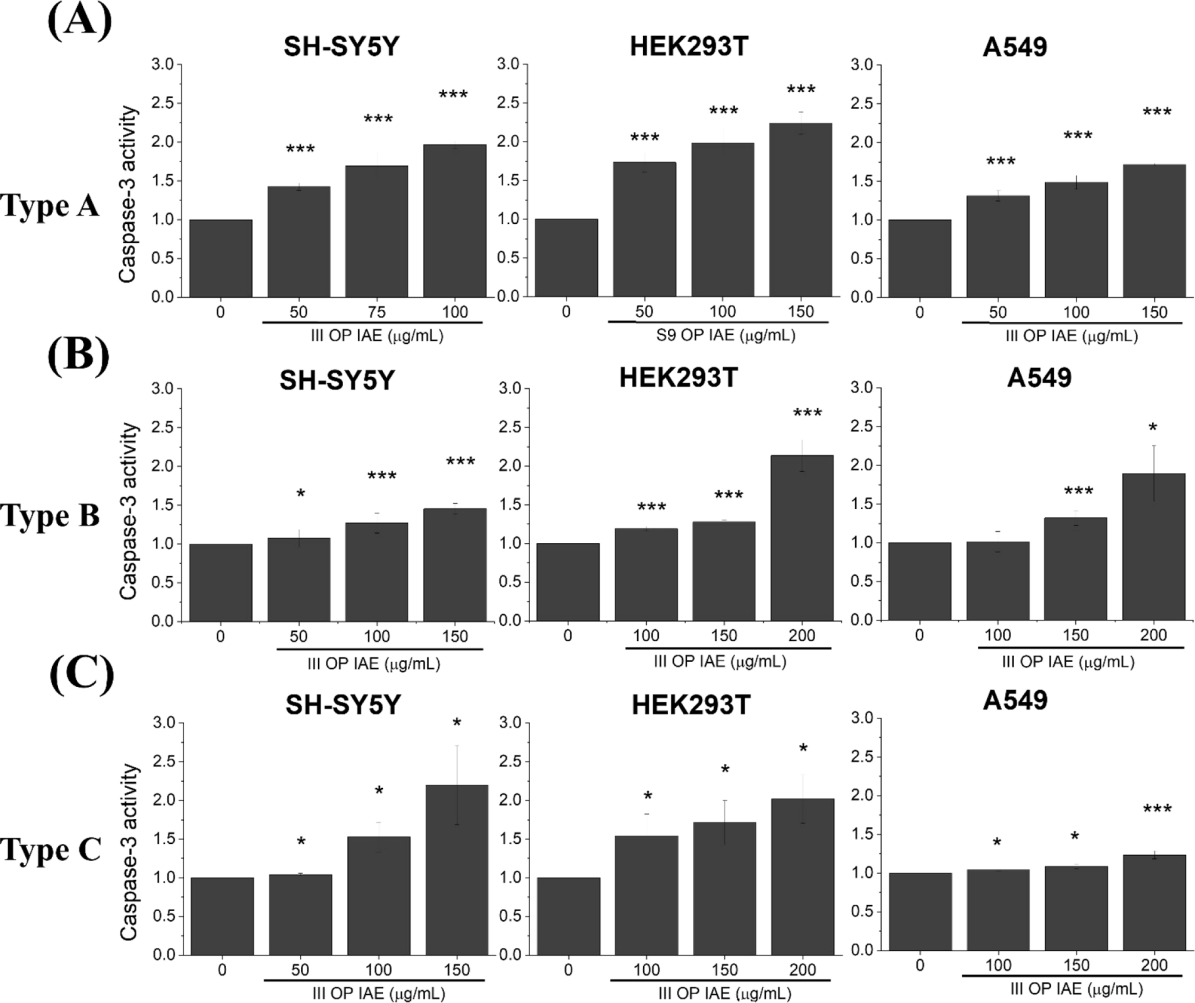
Caspase-3 activity in SH-SY5Y, HEK293T, and A549 cells following exposure to IAEs from (A) Type A incense, (B) Type B incense, and (C) Type C incense. I–IV denote MOUDI size fractions (see Figure 2A). Data represents mean ± SD from three independent experiments. Statistical significance: *p* < 0.05 (*), *p* < 0.01 (**), *p* < 0.005 (***).

**Figure 7:**
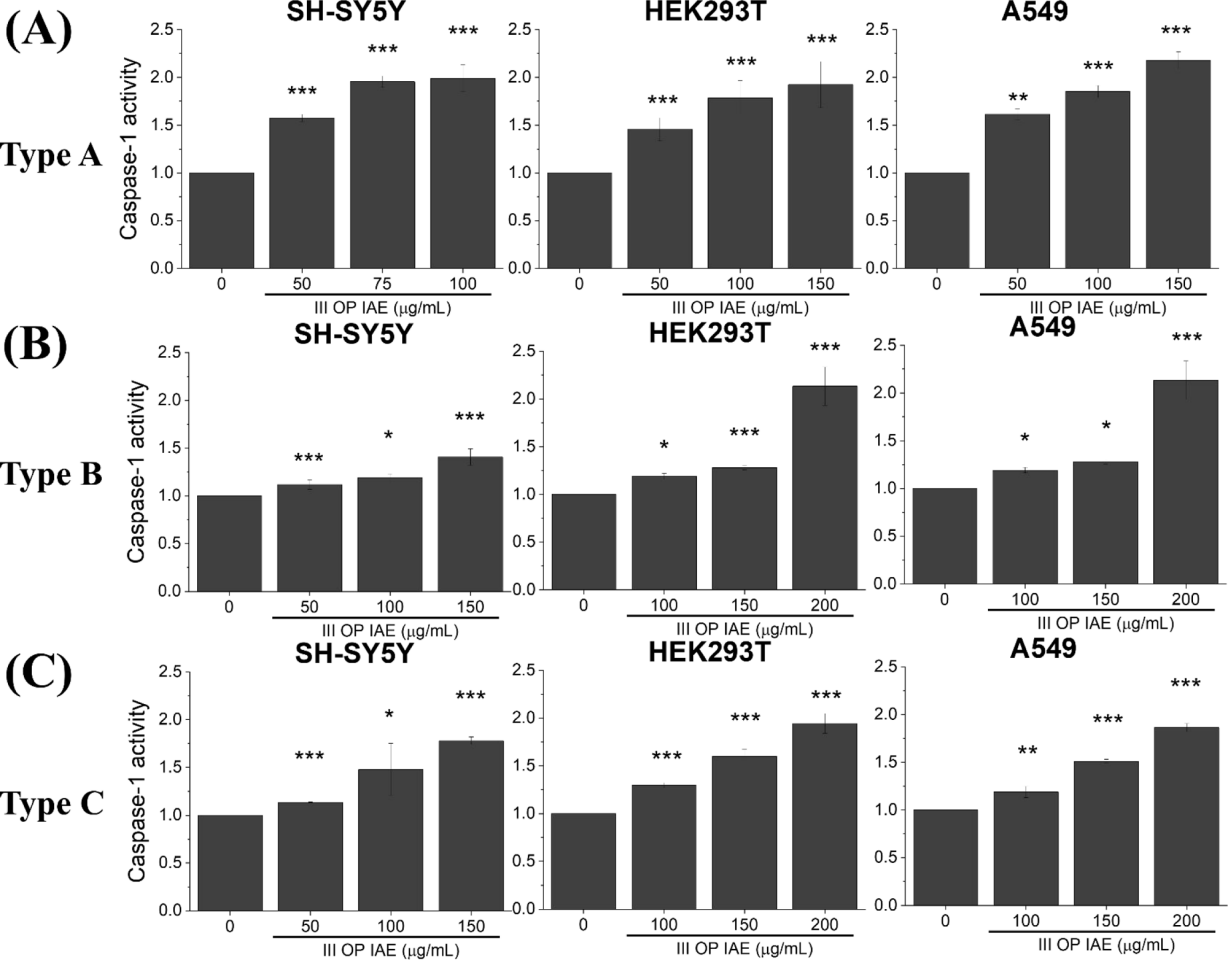
Caspase-1 activity in SH-SY5Y, HEK293T, and A549 cells following exposure to IAEs from (A) Type A incense, (B) Type B incense, and (C) Type C incense. I–IV denote MOUDI size fractions (see Figure 2A). Data represents mean ± SD from three independent experiments. Statistical significance: *p* < 0.05 (*), *p* < 0.01 (**), *p* < 0.005 (***).

**Figure 8:**
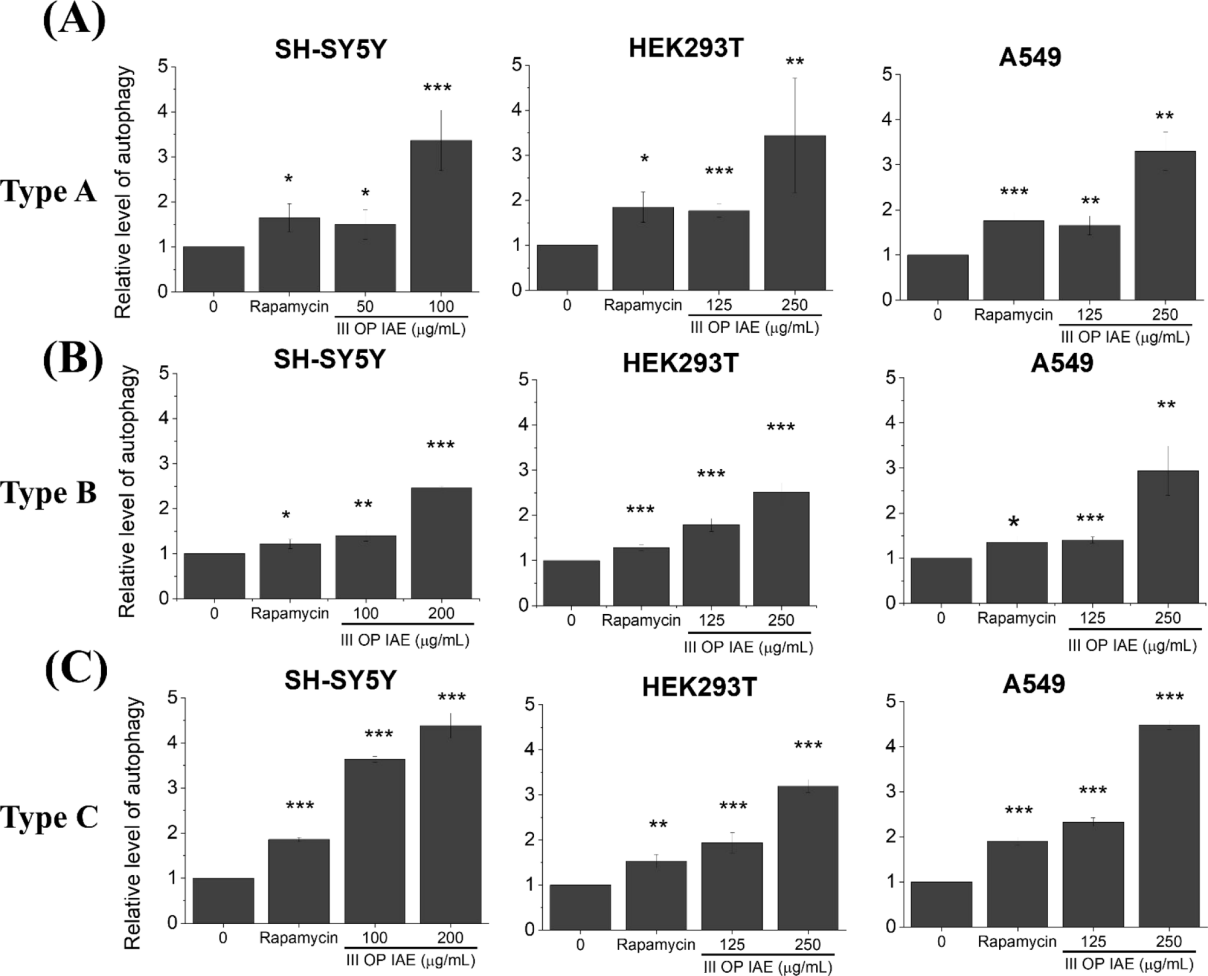
Autophagic activity in SH-SY5Y, HEK293T, and A549 cells following exposure to IAEs from (A) Type A incense, (B) Type B incense, and (C) Type C incense. I–IV denote MOUDI size fractions (see Figure 2A). Data represents mean ± SD from three independent experiments. Statistical significance: *p* < 0.05 (*), *p* < 0.01 (**), *p* < 0.005 (***).

Caspase-3 activity was measured to assess apoptotic responses (Figure 6). Two-way ANOVA revealed significant main effects of cell type (*p* < 0.001) and incense type (*p* < 0.0001) on caspase-3 activation, with no significant interaction between these factors. Post hoc analysis indicated that Incense Type A induced significantly higher caspase-3 activity than Types B and C across all cell lines. Additionally, SH-SY5Y and HEK293T cells exhibited higher caspase-3 activity compared to A549 cells, indicating greater susceptibility to apoptosis.

Pyroptotic signaling was assessed via caspase-1 activation (Figure 7). Two-way ANOVA revealed a significant main effect of incense type (*p* < 0.0001), while the main effect of cell type was not significant. Post hoc analysis demonstrated that Incense Type A induced significantly higher caspase-1 activity than Types B and C across all cell types, with no significant differences observed between Types B and C.

Autophagy activity was evaluated following exposure to the three incense types (Figure 8). Two-way ANOVA revealed significant main effects of cell type and incense type, as well as a significant interaction between these factors (all *p* < 0.0001). Overall, SH-SY5Y cells exhibited significantly higher autophagy activity than HEK293T and A549 cells even in a lower IAE concentration (100 μg/mL vs125 μg/mL, Figure 8). Unlike the caspase results, Incense Type C induced the strongest autophagic response, followed by Type A, while Type B elicited the weakest effect. Notably, autophagy induction by Incense Type C was most pronounced in SH-SY5Y cells, indicating a cell-type-specific response.

Across all end points, SH-SY5Y neuronal cells exhibited the highest sensitivity, consistent with their heightened susceptibility to oxidative and mitochondrial stress. Collectively, these results demonstrate that OP-IAE robustly engages apoptosis, pyroptosis, and autophagy pathways in an incense- and cell-type–dependent manner.

## 4 Discussion

Several limitations should be acknowledged. Extract-based exposure does not fully capture the dynamic processes of inhalation, particle deposition, clearance, or chronic low-dose exposure that occur in vivo, and direct extrapolation of in vitro concentrations to human exposure remains challenging due to the lack of standardized dose-conversion models for incense aerosols. Nevertheless, real-world monitoring consistently reports high concentrations of submicron and ultrafine particles during incense burning, supporting the environmental relevance of the particle fractions examined here. Although IAE-based treatment cannot fully recapitulate the complexity of long-term incense smoke inhalation, it enables controlled, size- and chemistry-resolved interrogation of aerosol constituents across human lung-, kidney-, and neuron-derived cell models. Using this approach, the present study systematically demonstrates that ultrafine and submicron incense aerosol fractions enriched in organic-phase constituents are the primary drivers of cellular toxicity, thereby providing mechanistic resolution beyond prior studies focused on bulk PM or PM_2_._5_.

A central and novel finding of this study is that incense aerosol extracts (IAEs) derived from particles smaller than 0.56 μm—particularly Fractions III and IV (<0.18 μm)—consistently exhibited significantly lower IC_50_ values than extracts from larger particles across all three cell types examined (Figure 3, Table 3). This pronounced size-dependent cytotoxicity reflects the intrinsic physicochemical properties of submicron aerosols, which possess substantially higher surface-area-to-mass ratios that facilitate adsorption, cellular uptake, and intracellular delivery of toxic constituents. Although similar size-dependent toxicity trends have been reported for ambient PM and cigarette smoke aerosols,^3,26–29^ our study extends these observations by directly linking size-segregated incense aerosols to quantitative cytotoxic outcomes using standardized aerosol generation, extraction, and dosing protocols. This approach addresses a critical methodological gap in incense toxicology and provides a reproducible platform for comparative risk assessment.

The elevated cytotoxic potency of fine and ultrafine IAEs is further supported by incense-type–specific differences in chemical composition. Our compositional analyses demonstrate that decreasing aerosol size is accompanied by enrichment in higher-molecular-weight PAHs and increasing chemical complexity, including toxic congeners such as benzo­[*a*]­pyrene, benzo­[*b*]­fluoranthene, and benzo­[*k*]­fluoranthene (Figure 2). Although these high-molecular-weight PAHs constitute a smaller fraction of total PAH mass, they disproportionately contribute to toxic equivalency, consistent with the markedly lower IC_50_ values observed for fine and ultrafine IAEs. Importantly, sandalwood-dominant incense (Type A) consistently exhibited the greatest cytotoxicity, followed by binchotan charcoal–dominant (Type C) and agarwood-dominant (Type B) incense (Figure 3). Previous emission studies have reported that sandalwood-based formulations typically possess higher hydrogen-to-carbon (H/C) ratios and elevated H/C ratios in solid fuels are known to favor smoldering combustion pathways that generate organic-rich aerosols enriched in PAHs and reactive organic species.^3,28^ This physicochemical characteristic plausibly explains the greater abundance and biological potency of the OP IAE derived from type A incense. The distinction between OP and WP IAE enables a more refined mechanistic interpretation (Figure 3). WP IAE generally produced minimal cytotoxicity and weak oxidative or mitochondrial responses, despite containing measurable metal ions. In contrast, OP IAE consistently drove strong effects across all end points, supporting the conclusion that lipophilic organic constituents rather than water-soluble components dominate incense aerosol toxicity under the tested conditions. This observation provides clear methodological justification for focusing on organic-phase fractions in mechanistic assays.

Oxidative stress emerged as a key mechanistic response linking particle size and composition to cellular injury. All three incense types increased intracellular ROS in a size- and potency-dependent manner, with ultrafine OP IAE eliciting the strongest responses and following the order Type A incense, followed by Type C incense, and then Type B incense (Figure 4). This pattern mirrors the relative organic and PAH burdens of these aerosols and directly supports the role of redox-active organic compounds and transition metals in incense-induced oxidative stress.^30–32^ While ROS generation by incense and cigarette aerosols has been widely reported, our size-resolved analysis demonstrates that ROS production is disproportionately driven by ultrafine organic-phase fractions, rather than by bulk particulate mass.^5,8,33^

Mitochondrial dysfunction was identified as a downstream consequence of oxidative stress. OP IAE from Fractions III and IV induced pronounced loss of MMP and depletion of intracellular ATP across all three cell models (Figure 5). The concordant decline in MMP and ATP provides experimental evidence that mitochondrial impairment represents a convergent target of ultrafine incense aerosols, consistent with prior observations for PM_2_._5_ ^34,35^ and cigarette smoke^36,37^ but demonstrated here for incense aerosols in a size- and phase-specific manner. Importantly, Type A incense aerosol extracts induced significantly greater mitochondrial disruption than Type B and Type C extracts, consistent with their lower IC_50_ values and higher levels of ROS production.

Downstream of oxidative and mitochondrial stress, IAE exposure activated multiple programmed cell death pathways. Fraction III OP IAE, selected due to their abundance and strong biological activity, induced apoptosis, pyroptosis, and autophagy across all cell types (Figures 6–8). Caspase-3 and caspase-1 activation directly link mitochondrial dysfunction and oxidative stress to apoptotic and inflammasome-associated cell death, respectively, while increased autophagic activity suggests engagement of stress-adaptive responses that may precede terminal outcomes.^38–43^ Although similar pathways have been reported for cigarette smoke,^44–46^ our data demonstrate for the first time that incense aerosols engage multiple programmed cell death mechanisms in a size-resolved manner, underscoring their complex cytotoxic potential.

Comparative analysis across cell models revealed marked differential susceptibility. SH-SY5Y neuronal cells consistently exhibited the strongest responses across cytotoxicity, oxidative stress, mitochondrial disruption, and programmed cell death assays, followed by A549 lung epithelial cells and HEK293T kidney-derived cells. This heightened neuronal sensitivity likely reflects increased metabolic demand and vulnerability to mitochondrial perturbation, supporting emerging epidemiological associations between incense exposure and neurotoxicity. These findings highlight the importance of incorporating nonpulmonary cell models when evaluating indoor aerosol hazards.

## 5 Conclusion

In summary, this study delineates a clear size-dependent mechanistic pathway by which IAE induces multiorgan cytotoxicity. Our findings demonstrate that the organic-phase fractions (diameters <0.18 μm) act as the primary drivers of cellular injury, owing to their high content of lipophilic toxicants. Upon internalization, these particles trigger a rapid escalation in intracellular ROS, which subsequently precipitates mitochondrial dysfunction, evidenced by the collapse of MMP and critical ATP depletion. This bioenergetic failure serves as the nexus for activating a complex network of programmed cell death, including apoptosis, pyroptosis, and autophagy (Figure 9). The conservation of this ROS–mitochondrial axis across pulmonary, renal, and especially vulnerable neuronal cell types underscores the systemic health risks posed by incense smoke exposure and provides a molecular framework for future toxicological assessments and public health interventions. These findings demonstrate that reduced visible smoke does not necessarily equate to reduced biological risk and underscore the importance of size- and chemistry-resolved assessment in indoor air-quality evaluation. Improved ventilation, reduced burn duration, and formulation standards targeting ultrafine organic emissions may therefore be critical strategies for mitigating incense-related health risks.

**Figure 9:**
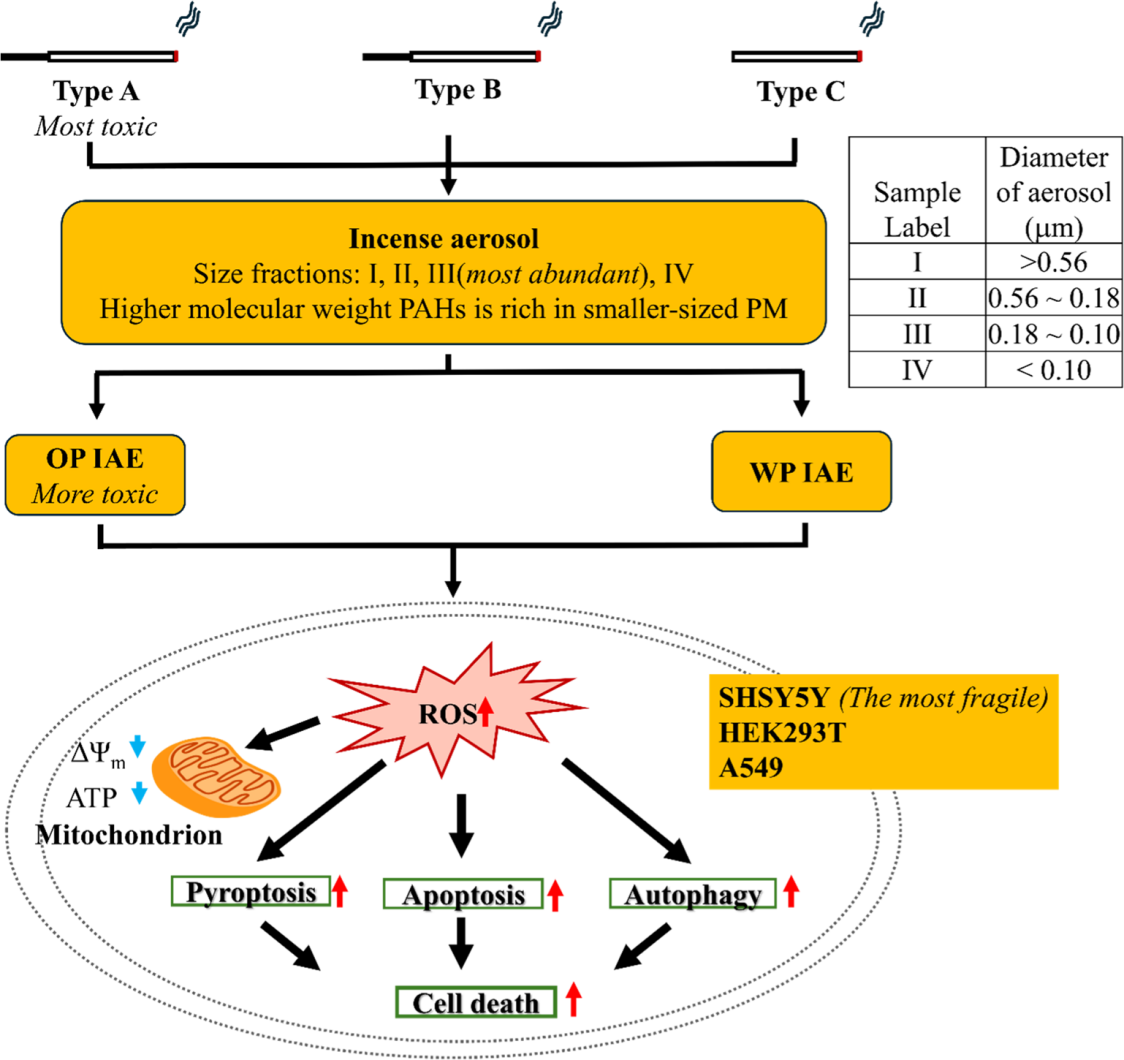
Summary mechanism of IAE-induced cell death.

## Supplementary Material



## Notes

Supporting Information includes the following information: Size-dependent OP and WP IAE influence on living cells verified by MTT assay (Figures S1–S3) (PDF)

## Abbreviations

IAE: incense aerosol extract
CAE: cigarette aerosol extract
MMP: mitochondrial membrane potential
OP: organic phase
PAHs: polycyclic aromatic hydrocarbons
PM: particulate matter
ROS: reactive oxygen species
WP: water-soluble phase
SVOCs: semivolatile organic compounds
APS: aerodynamics particle sizer
SMPS: scanning mobility particle sizer
**GC**-**MS**/**MS**: gas chromatography–tandem mass spectrometry
**ICP**-**MS**: inductively coupled plasma mass spectrometry
MOUDI: Micro-Orifice Uniform Deposit Impactor

## References

[1] Apte K., Salvi S. (2016). Household
air pollution and its effects on health. F1000.

[2] Yang T. T., Chen C. C., Lin J. M. (2005). Effect of air flow
on emission of
smoldering incense. Bull. Environ. Contam. Toxicol..

[3] Yang T. T., Ho S. C., Chuang L. T., Chuang H. C., Li Y. T., Wu J. J. (2017). Characterization
of particulate-phase polycyclic aromatic hydrocarbons
emitted from incense burning and their bioreactivity in RAW264.7 macrophage. Environ. Pollut..

[4] Lee S. C. W. B., Wang B. (2004). Characteristics of emissions of air
pollutants from
burning of incense in a large environmental chamber. Atmos. Environ..

[5] Chuang H. C., Jones T., Chen Y., Bell J., Wenger J., BeruBe K. (2011). Characterisation of
airborne particles and associated
organic components produced from incense burning. Anal. Bioanal. Chem..

[6] Lui K. H., Bandowe B. A. M., Ho S. S. H., Chuang H. C., Cao J. J., Chuang K. J., Lee S. C., Hu D., Ho K. F. (2016). Characterization
of chemical components and bioreactivity of fine particulate matter
(PM2.5) during incense burning. Environ. Pollut..

[7] Tung J. C., Huang W. C., Yang J. C., Chen G. Y., Fan C. C., Chien Y. C., Lin P. S., Candice Lung S. C., Chang W. C. (2017). Auramine O, an incense smoke ingredient,
promotes lung
cancer malignancy. Environ. Toxicol..

[8] Wang B., Lee S. C., Ho K. F., Kang Y. M. (2007). Characteristics
of emissions of air pollutants from burning of incense in temples,
Hong Kong. Sci. Total Environ..

[9] Lin T. C., Krishnaswamy G., Chi D. S. (2008). Incense smoke: clinical, structural
and molecular effects on airway disease. Clin.
Mol. Allergy.

[10] Lin T. C., Chang F. H., Hsieh J. H., Chao H. R., Chao M. R. (2002). Characteristics
of polycyclic aromatic hydrocarbons and total suspended particulate
in indoor and outdoor atmosphere of a Taiwanese temple. J. Hazard. Mater..

[11] Phairuang W., Hongtieab S., Suwattiga P., Furuuchi M., Hata M. (2022). Atmospheric
Ultrafine Particulate Matter (PM0.1)-Bound Carbon Composition in Bangkok,
Thailand. Atmosphere.

[12] Phairuang W., Piriyakarnsakul S., Inerb M., Hongtieab S., Thongyen T., Chomanee J., Boongla Y., Suriyawong P., Samae H., Chanonmuang P. (2023). Ambient Nanoparticles
(PM0.1) Mapping in Thailand. Atmosphere.

[13] Suan
Tial M. K., Kyi N. N., Amin M., Hata M., Furuuchi M., Putri R. M., Paluang P., Suriyawong P., Phairuang W. (2024). Size-fractionated carbonaceous particles and climate
effects in the eastern region of Myanmar. Particuology.

[14] Cheng Y. S., W E.
B., Yu C. C., Hung I. F. (1995). Incense Smoke: Characterization
and Dynamics in Indoor Environments. Aerosol
Sci. Technol..

[15] Akin F. J., Snook M. E., Severson R. E., Chamberlain W. J., Walters D. B. (1976). Identification of polynuclear aromatic
hydrocarbons
in cigarette smoke and their importance as tumorigens. J. Natl. Cancer Inst.

[16] Yamamoto N., Kan-o K., Tatsuta M., Ishii Y., Ogawa T., Shinozaki S., Fukuyama S., Nakanishi Y., Matsumoto K. (2021). Incense smoke-induced oxidative stress disrupts tight
junctions and bronchial epithelial barrier integrity and induces airway
hyperresponsiveness in mouse lungs. Sci. Rep..

[17] Chuang H. C., Jones T., Chen T. T., BeruBe K. (2013). Cytotoxic effects of
incense particles in relation to oxidative stress, the cell cycle
and F-actin assembly. Toxicol. Lett..

[18] Mannix R. C., Nguyen K. P., Tan E. W., Ho E. E., Phalen R. F. (1996). Physical
characterization of incense aerosols. Sci. Total
Environ..

[19] Hussain T., Alamery S., Dikshit G., Mohammed A. A., Naushad S. M., Alrokayan S. (2019). Incense smoke
exposure augments systemic oxidative
stress, inflammation and endothelial dysfunction in male albino rats. Toxicol. Mech. Methods.

[20] Al-Attas O. S., Hussain T., Ahmed M., Al-Daghri N., Mohammed A. A., De Rosas E., Gambhir D., Sumague T. S. (2015). Ultrastructural
changes, increased oxidative stress, inflammation, and altered cardiac
hypertrophic gene expressions in heart tissues of rats exposed to
incense smoke. Environ. Sci. Pollut Res. Int..

[21] Hussain T., Al-Attas O. S., Alrokayan S. A., Ahmed M., Al-Daghri N. M., Al-Ameri S., Pervez S., Dewangan S., Mohammed A., Gambhir D. (2016). Deleterious
effects of incense smoke exposure on kidney
function and architecture in male albino rats. Inhal. Toxicol..

[22] Sudhakaran G., Ramamurthy K., Dhaareshwar V. N., Rajagopal R., Alfarhan A., Arockiaraj J. (2024). Neurotoxic
and developmental effects
of scented incense stick smoke: Network toxicology and zebrafish model
study. Toxicol. Lett..

[23] Chi M. C., Lin Z. C., Lee C. W., Huang C. C., Peng K. T., Lin C. M., Lee H. C., Fang M. L., Chiang Y. C. (2023). Tanshinone
IIA suppresses burning incense-induced oxidative stress and inflammatory
pathways in astrocytes. Ecotoxicol. Environ.
Saf..

[24] Lee C. W., Vo T. T. T., Wee Y., Chiang Y. C., Chi M. C., Chen M. L., Hsu L. F., Fang M. L., Lee K. H., Guo S. E. (2021). The
Adverse Impact of Incense Smoke on Human Health:
From Mechanisms to Implications. J. Inflamm
Res..

[25] Yadav V. K., Malik P., Tirth V., Khan S. H., Yadav K. K., Islam S., Choudhary N., Inwati G. K., Arabi A., Kim D. H. (2022). Health
and Environmental Risks of Incense Smoke: Mechanistic
Insights and Cumulative Evidence. J. Inflamm
Res..

[26] Shen Y. X., Lee P. S., Wang C. C., Teng M. C., Huang J. H., Fan H. F. (2024). Exploring the Cellular
Impact of Size-Segregated Cigarette
Aerosols: Insights into Indoor Particulate Matter Toxicity and Potential
Therapeutic Interventions. Chem. Res. Toxicol..

[27] Shen Y. X., Lee P. S., Teng M. C., Huang J. H., Wang C. C., Fan H. F. (2024). Influence of Cigarette
Aerosol in Alpha-Synuclein Oligomerization
and Cell Viability in SH-SY5Y: Implications for Parkinson’s
Disease. ACS Chem. Neurosci..

[28] Yang T. T., Lin S. T., Lin T. S., Hong W. L. (2012). Characterization
of polycyclic aromatic hydrocarbon emissions in the particulate phase
from burning incenses with various atomic hydrogen/carbon ratios. Sci. Total Environ..

[29] Zhou R., An Q., Pan X. W., Yang B., Hu J., Wang Y. H. (2015). Higher
cytotoxicity and genotoxicity of burning incense than cigarette. Environ. Chem. Lett..

[30] Zhao S.-M., Liu W.-X., Miao Q.-Y., Li J.-M., Sun L.-M., Wu S.-P. (2025). Emissions characteristics of polycyclic
aromatic hydrocarbons, water-soluble
heavy metals, and oxidative potential from indoor non-energy combustion
sources. Environ. Sci. Pollut. Res..

[31] Chuang H. C., Jones T. P., Lung S. C., BéruBé K. A. (2011). Soot-driven
reactive oxygen species formation from incense burning. Sci. Total Environ..

[32] Lui K. H., Bandowe B. A. M., Ho S. S. H., Chuang H.-C., Cao J.-J., Chuang K.-J., Lee S. C., Hu D., Ho K. F. (2016). Characterization
of chemical components and bioreactivity of fine particulate matter
(PM2.5) during incense burning. Environ. Pollut..

[33] Song K., Tang R., Li A., Wan Z., Zhang Y., Gong Y., Lv D., Lu S., Tan Y., Yan S. (2023). Particulate organic emissions from incense-burning
smoke: Chemical compositions and emission characteristics. Sci. Total Environ..

[34] Gao M., Liang C., Hong W., Yu X., Zhou Y., Sun R., Li H., Huang H., Gan X., Yuan Z. (2022). Biomass-related PM2.5 induces mitochondrial
fragmentation and dysfunction
in human airway epithelial cells. Environ. Pollut..

[35] An Z., Liu G., Shen L., Qi Y., Hu Q., Song J., Li J., Du J., Bai Y., Wu W. (2024). Mitochondrial dysfunction
induced by ambient fine particulate matter and potential mechanisms. Environ. Res..

[36] van
der Toorn M., Rezayat D., Kauffman H. F., Bakker S. J., Gans R. O., Koeter G. H., Choi A. M., van Oosterhout A. J., Slebos D. J. (2009). Lipid-soluble components in cigarette smoke induce
mitochondrial production of reactive oxygen species in lung epithelial
cells. Am. J. Physiol Lung Cell Mol. Physiol.

[37] Liang L., Wang L., Jonker M. R., Kosse W., Jellema P. G., Rots M. G., Heijink I. H. (2025). The effects
of cigarette smoke extract
on mitochondrial function, mitochondrial gene expression and mitochondrial
DNA methylation in airway epithelial cells. Epigenet. Commun..

[38] Green D. R., Llambi F. (2015). Cell Death Signaling. Cold Spring
Harb Perspect Biol..

[39] Andon F. T., Fadeel B. (2013). Programmed cell death:
molecular mechanisms and implications
for safety assessment of nanomaterials. Acc.
Chem. Res..

[40] Miao E. A., Rajan J. V., Aderem A. (2011). Caspase-1-induced
pyroptotic cell
death. Immunol. Rev..

[41] Schroder K., Tschopp J. (2010). The inflammasomes. Cell.

[42] Chen X., He W. T., Hu L., Li J., Fang Y., Wang X., Xu X., Wang Z., Huang K., Han J. (2016). Pyroptosis is driven by non-selective
gasdermin-D pore and its morphology
is different from MLKL channel-mediated necroptosis. Cell Res..

[43] Shi J., Zhao Y., Wang K., Shi X., Wang Y., Huang H., Zhuang Y., Cai T., Wang F., Shao F. (2015). Cleavage of GSDMD by inflammatory caspases determines pyroptotic
cell death. Nature.

[44] Son E. S., Kim S. H., Ryter S. W., Yeo E. J., Kyung S. Y., Kim Y. J., Jeong S. H., Lee C. S., Park J. W. (2018). Quercetogetin
protects against cigarette smoke extract-induced apoptosis in epithelial
cells by inhibiting mitophagy. Toxicol. In Vitro.

[45] Zhang M.-Y., Jiang Y.-X., Yang Y.-C., Liu J.-Y., Huo C., Ji X.-L., Qu Y.-Q. (2021). Cigarette
smoke extract induces pyroptosis
in human bronchial epithelial cells through the ROS/NLRP3/caspase-1
pathway. Life Sci..

[46] Chen Z. H., Lam H. C., Jin Y., Kim H. P., Cao J., Lee S. J., Ifedigbo E., Parameswaran H., Ryter S. W., Choi A. M. (2010). Autophagy protein microtubule-associated
protein 1 light chain-3B (LC3B) activates extrinsic apoptosis during
cigarette smoke-induced emphysema. Proc. Natl.
Acad. Sci. U. S. A..

