# Supplementary material for: Size-Segregated Incense Aerosols Drive ROS–Mitochondrial Dysfunction and Programmed Cell Death Across Human Cell Types
Source: Chem Res Toxicol. 2026 Feb 9;39(3):427–41. doi: 10.1021/acs.chemrestox.6c00020 (PMC12997252; doi:10.1021/acs.chemrestox.6c00020)
Supplement: Supplementary file 1 [file tx6c00020_si_001.pdf]

Supporting information

Size-Segregated Incense Aerosols Drive ROS–Mitochondrial Dysfunction and Programmed Cell Death Across Human Cell Types

Yi-En Tseng[1,2,+], Ming-Chu Teng [1,2,+], Yu-Siou Huang[1,2,+], Padhmavathi Selvam[1,2,+], Chia-Hsuan Pan[2, 3], Yuan-Pin Chang[2], Chia C. Wang[2,3*], Hsiu-Fang Fan[1,2,3*]

[1] Institute of Medical Science and Technology, National Sun Yat-sen University, Kaohsiung, 804, Taiwan

[2] Department of Chemistry, National Sun Yat-sen University, Kaohsiung, 804, Taiwan

[3] Aerosol Science Research Center, National Sun Yat-sen University, Kaohsiung, 804, Taiwan

**** Correspondence can be sent to***

HFF *(bendyfan@imst.nsysu.edu.tw), and* ***CCW*** *(chiawang@mail.nsysu.edu.tw)*

To be submitted to Chemical Research in Toxicology

+ These authors have equal contributions to this work

Table of Contents

Figure S1. Cytotoxicity curves used for IC_{50} determination for size-fractionated Type A incense aerosols……………………………………………………………………….S1-S2

Figure S2. Cytotoxicity curves used for IC_{50} determination for size-fractionated Type B incense aerosols……………………………………………………………………….S3-S4

Figure S3. Cytotoxicity curves used for IC_{50} determination for size-fractionated Type C incense aerosols……………………………………………………………………….S5-S6

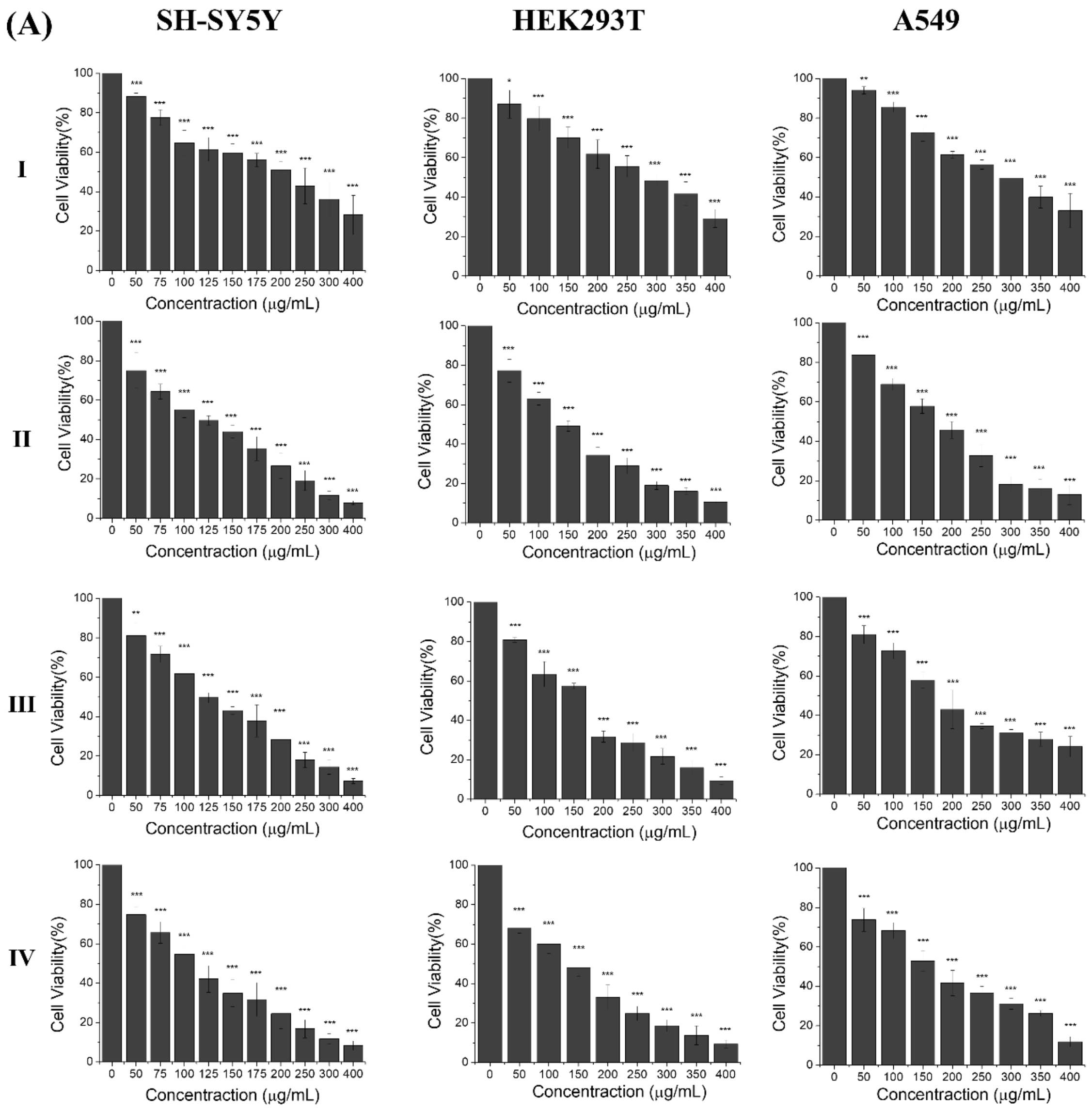

(A)
SH-SY5Y
HEK293T
A549
I
II
III
IV
Cell Viability(%)
Concentraction (μg/mL)

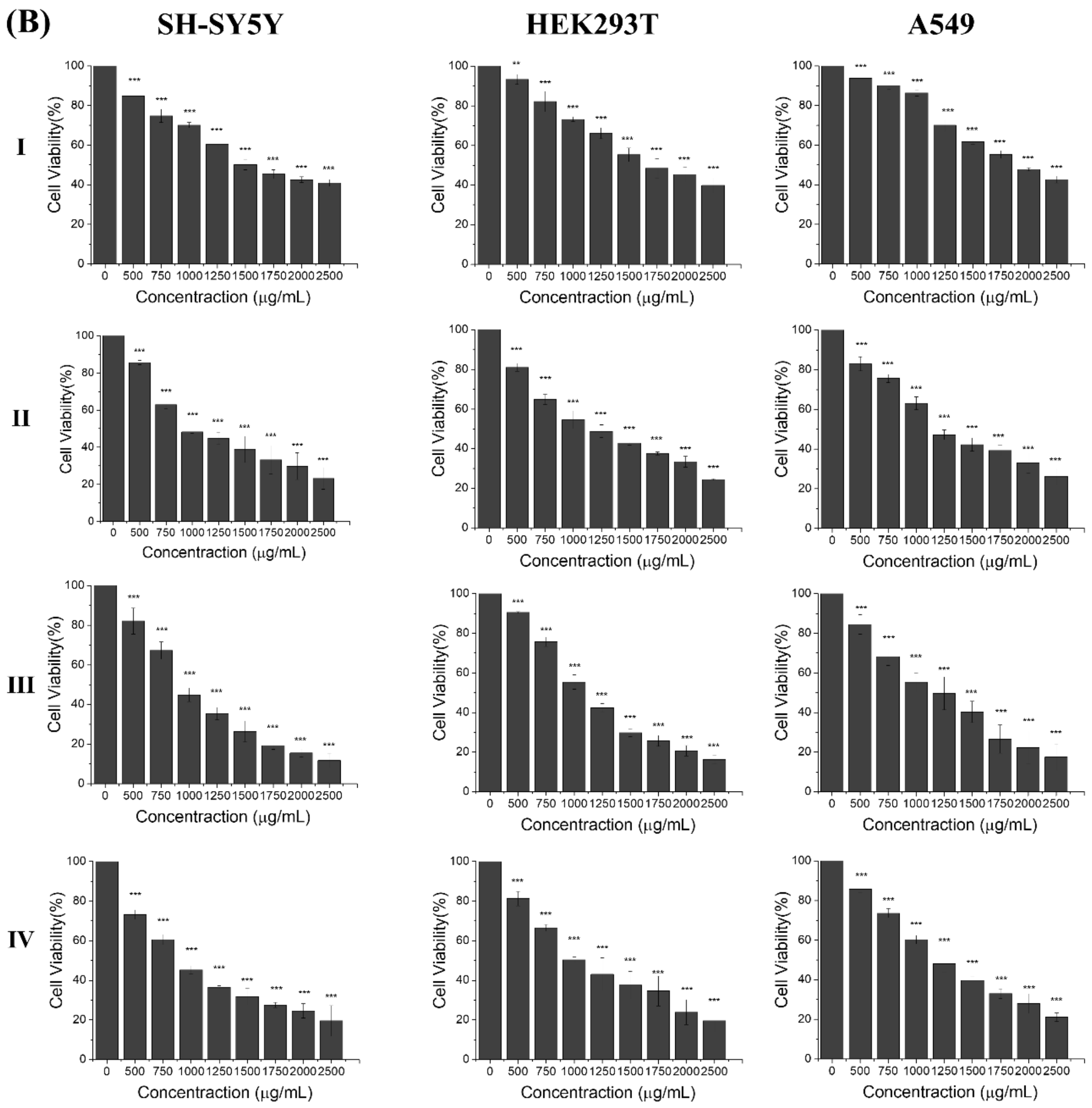

Figure S1 Size-dependent Type A incense (A) OP IAE and (B) WP IAE influence on living cells verified by MTT assay. Dose response curve of IAE in SH-SY5Y, HEK293T, and A549 cell lines using the MTT assays. *, **, and *** represent significant differences (* = $p < 0.05$), (** = $p < 0.01$) and (*** = $p < 0.005$). The numbers (N) indicate the number of experiments conducted in each condition. I~IV indicates the size of incense aerosol obtained with MOUDI listed in Fig. 2A.

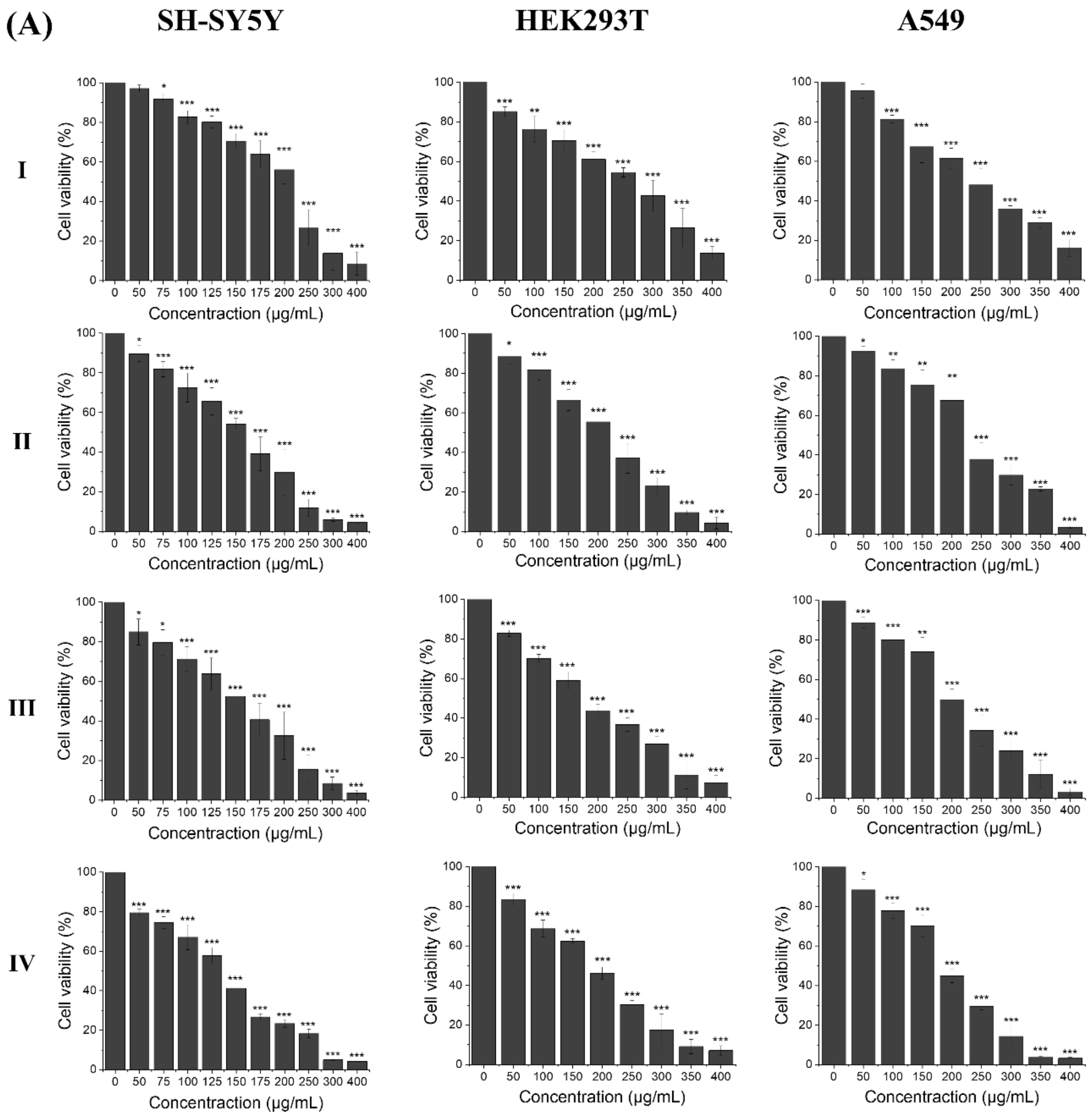
(A)
SH-SY5Y
HEK293T
A549
I
II
III
IV
Cell vaibility (%)
Cell viability (%)
Concentraction (μg/mL)
Concentration (μg/mL)

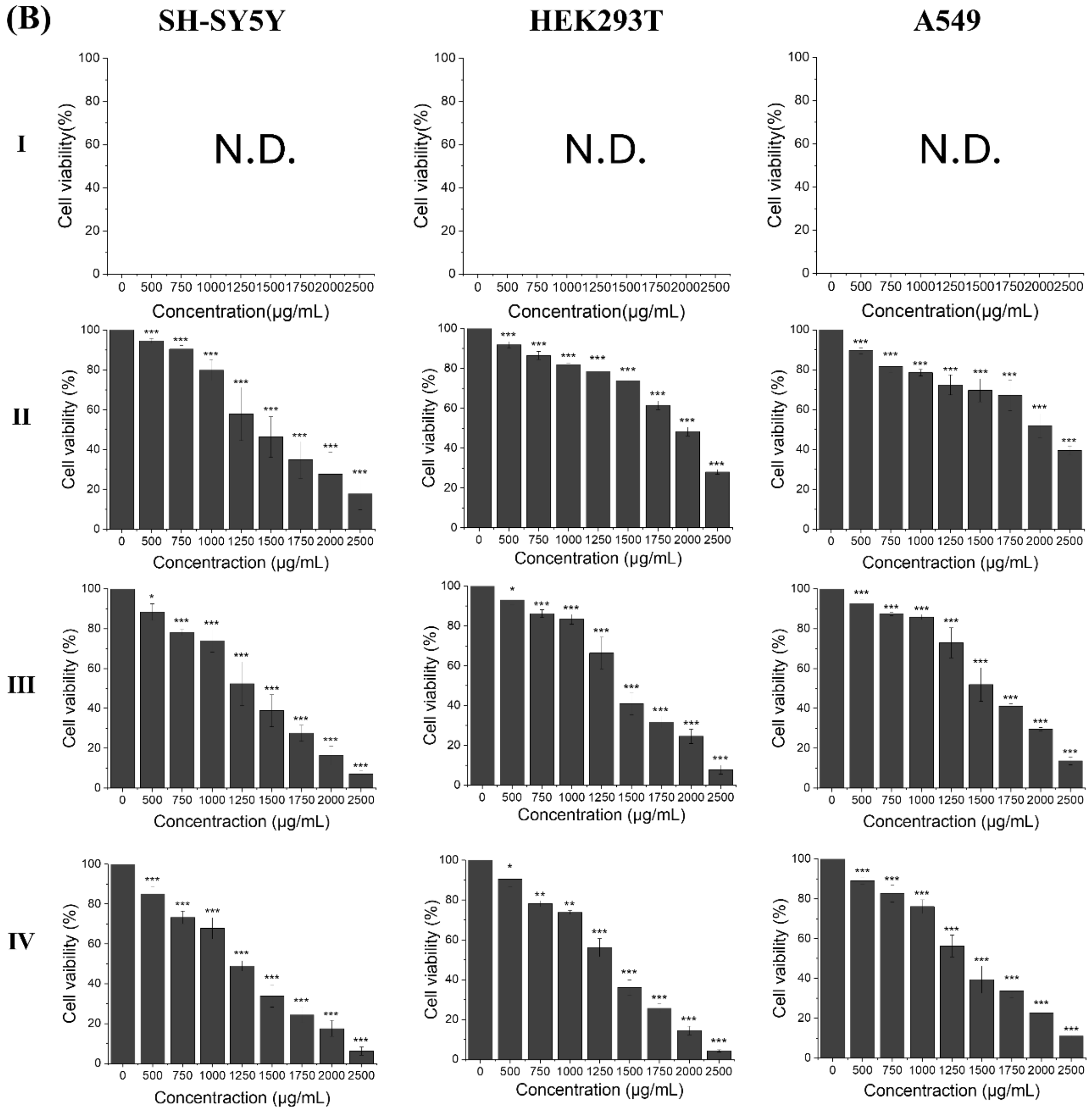

Figure S2 Size-dependent Type B incense (A) OP IAE and (B) WP IAE influence on living cells verified by MTT assay. The dose response curve of IAE in SH-SY5Y, HEK293T, and A549 cell lines using the MTT assays. *, **, and *** represent significant differences (* = p < 0.05), (** = p < 0.01) and (*** = p < 0.005). The numbers (N) indicate the number of experiments conducted in each condition. I~IV indicates the size of incense aerosol obtained with MOUDI listed in Fig. 2A.

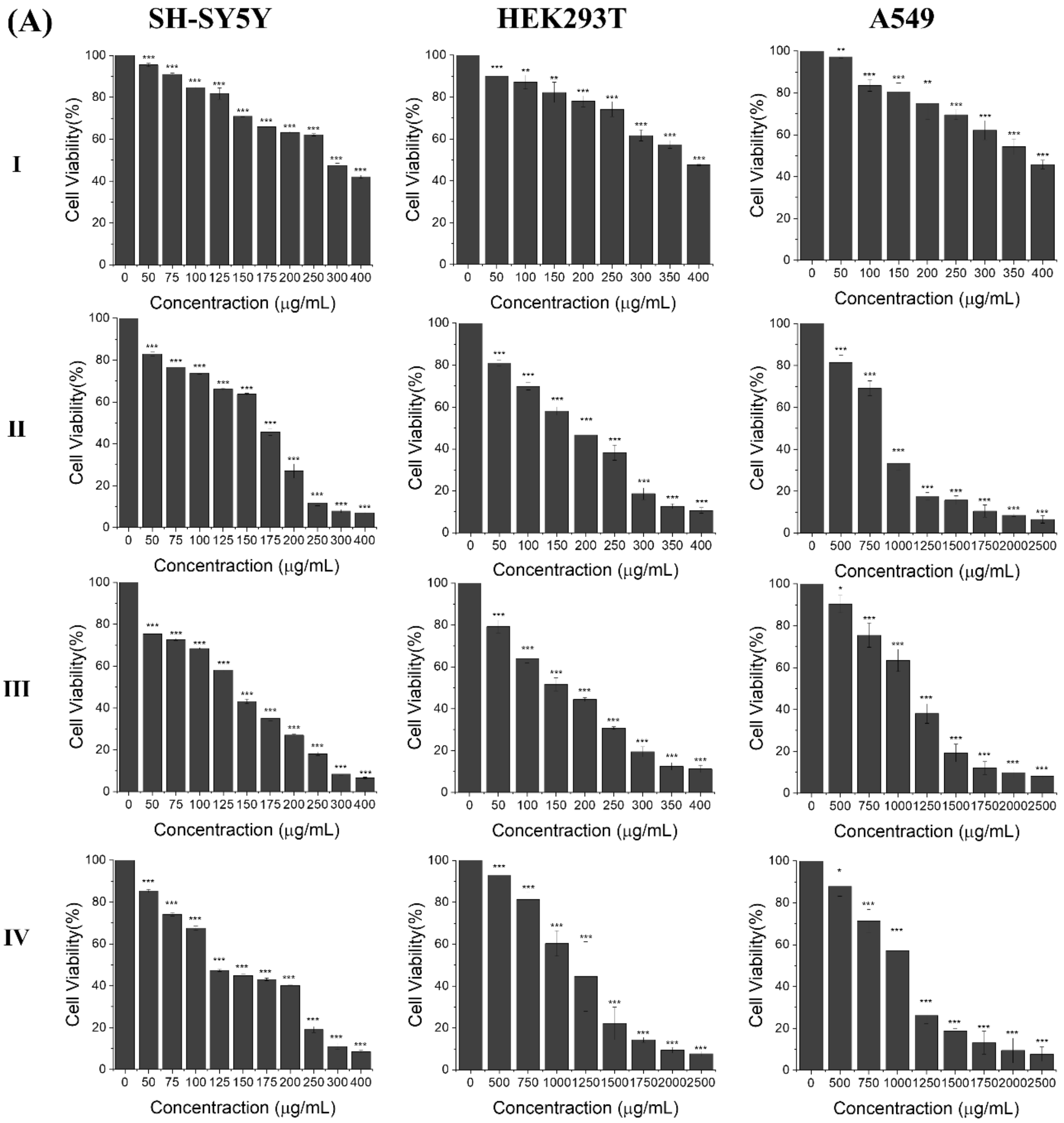
(A)
SH-SY5Y
HEK293T
A549
I
II
III
IV
Cell Viability(%)
Concentraction (μg/mL)

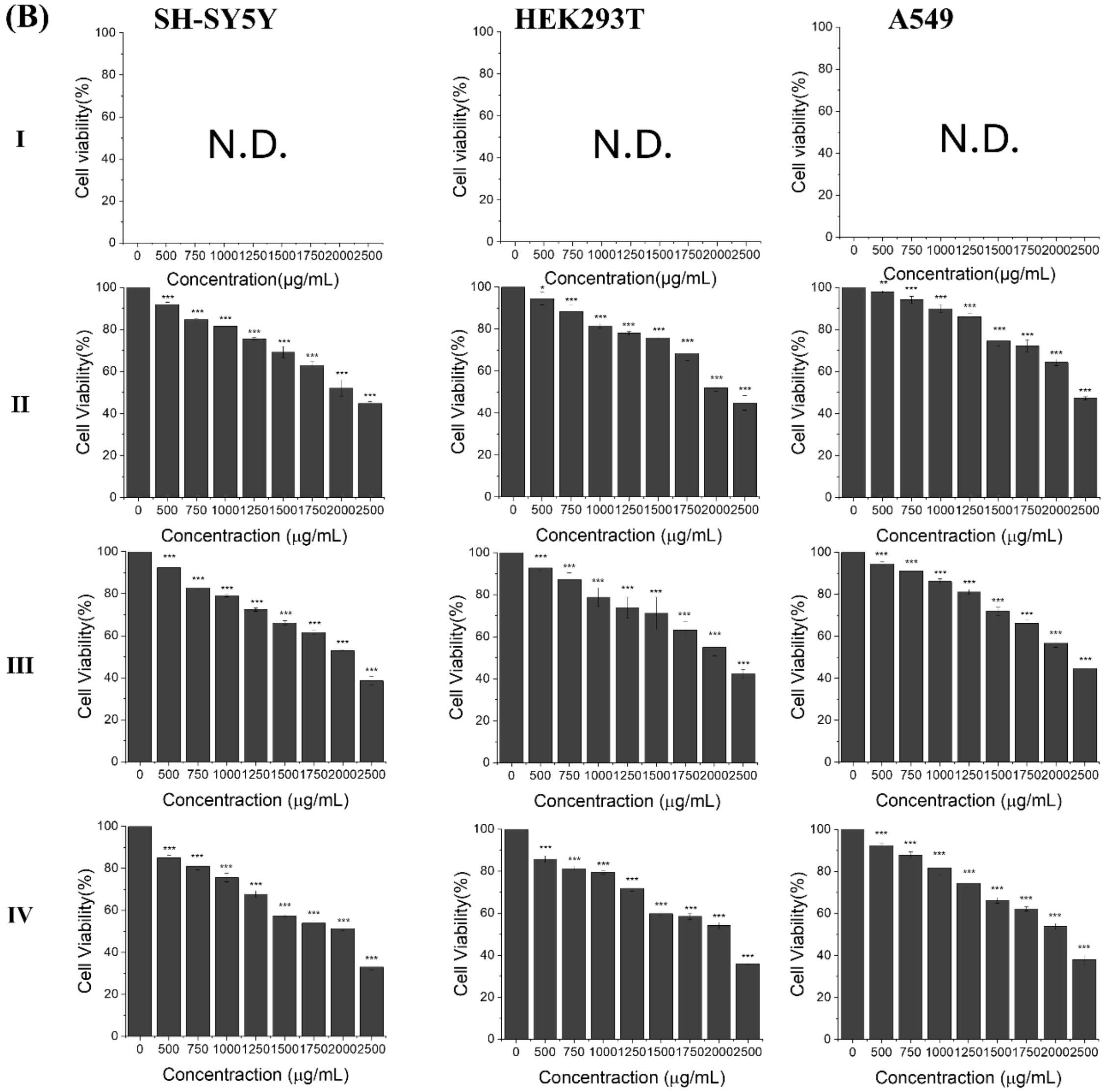

Figure S3 Size-dependent Type C incense (A) OP IAE and (B) WP IAE influence on living cells verified by MTT assay. Dose response curve of IAE in SH-SY5Y, HEK293T, and A549 cell lines using the MTT assays. *, **, and *** represent significant differences (* = $p < 0.05$), (** = $p < 0.01$) and (*** = $p < 0.005$). The numbers (N) indicate the number of experiments conducted in each condition. I~IV indicates the size of incense aerosol obtained with MOUDI listed in Fig. 2A.
